# Productive Ecosystems and the arrow of development

**DOI:** 10.1038/s41467-021-21689-0

**Published:** 2021-03-05

**Authors:** Neave O’Clery, Muhammed Ali Yıldırım, Ricardo Hausmann

**Affiliations:** 1 Centre for Advanced Spatial Analysis, University College London, London, UK; 2 Mathematical Institute, University of Oxford, Oxford, UK; 3 Growth Lab, Center for International Development, Harvard University, Cambridge, MA USA; 4 Koç University, Sarıyer/İstanbul, Turkey; 5 Harvard Kennedy School, Cambridge, MA USA; 6 Santa Fe Institute, Santa Fe, NM USA

**Keywords:** Environmental economics, Industry, Developing world, Economics

## Abstract

Economic growth is associated with the diversification of economic activities, which can be observed via the evolution of product export baskets. Exporting a new product is dependent on having, and acquiring, a specific set of capabilities, making the diversification process path-dependent. Taking an agnostic view on the identity of the capabilities, here we derive a probabilistic model for the directed dynamical process of capability accumulation and product diversification of countries. Using international trade data, we identify the set of pre-existing products, the product Ecosystem, that enables a product to be exported competitively. We construct a directed network of products, the Eco Space, where the edge weight corresponds to capability overlap. We uncover a modular structure, and show that low- and middle-income countries move from product communities dominated by small Ecosystem products to advanced (large Ecosystem) product clusters over time. Finally, we show that our network model is predictive of product appearances.

## Abstract (web summary)

As countries experience economic growth, diversification of economic activities may occur. Here, the authors develop a probabilistic model to examine the diversification of economic activities and how countries may move from small ecosystem products to advanced product clusters over time.

## Introduction

There is strong evidence that as countries experience economic growth, they change what they do and undergo structural transformation via diversification of their economic activities^1,2^ by increasing the number of industries that they have comparative advantage in. Emergence of a particular industry in a country depends on the availability of different combinations of capabilities, including various factors like capital, labour, and productive knowledge^3–5^. From this viewpoint, countries grow as they acquire productive knowledge and/or capabilities, and learn to combine these complementary capabilities in order to move into new economic activities. Hence, industrialisation is mostly a path-dependent process, whereby the appearance of new industries and economic activities is conditional on having or acquiring the relevant capabilities and know-how^3–8^.

Drawing up an exhaustive list of capabilities and/or the productive knowledge required for an industry is challenging. For instance, for a country to develop the fresh-cut flower industry, it requires capabilities, such as cold storage facilities, airports, irrigation systems, suitable climate, efficient customs, a good business environment as well as knowledge embedded in its farmers, botanic experts, engineers, logistic specialists, marketing professionals, bureaucrats and business executives to name but a few. This list is by no means exhaustive and the listed components might not be independent of each other. Since these capabilities are difficult to observe and measure, we do not try to uncover their identities and seek to quantify their existence drawing inspiration from biology and, in particular, the study of genetics. In genetics, observed phenotypes are the result of genotypes encoded in genetic material. Mendel, in his landmark study^9^, recorded the phenotypes present in successive generations of peas without directly observing the underlying genes and DNA structure. Hence, valuable information can be gathered by observing the phenotypic traits of individuals when the underlying genetic structure is unknown. Furthermore, by observing which phenotypic traits often co-occur in individuals, or which traits often follow each other, we can uncover genetic relationships or distances^10^. The genetic distance between phenotypes is relevant, for example, to inferring relationships between diseases^11^.

Here we take an agnostic view on the identity of the capabilities and we derive a probabilistic model to describe the directed dynamic process of capability accumulation and product diversification of countries. We use the presence and appearance of industries in countries (phenotypes) to infer capability and know-how-based (genotypic) relationships between industries. Using our genetics-inspired industry capability distance, and modelling industrial diversification as a process by which countries accumulate capabilities and move into new industries that share existing capabilities, we can predict the emergence of new industries.

A number of well-established models of economics can be interpreted from a genetic or phenotypic perspective. For instance, standard trade theories first proposed by Ricardo^12^, and Hecksher and Ohlin^13^ take complementary approaches which can be thought as phenotypic and genotypic stances, respectively, to explain trade patterns between countries. For example, a recent and celebrated version of the Ricardian model developed by Eaton and Kortum^14^ proposes that technological differences across countries, and the relative evolution of productivity across exports, determines the pattern of production in the world. These authors do not seek to uncover the causes behind the observed pattern, hence implicitly taking a phenotypic view of the international trade. On the other hand, the Hecksher–Ohlin model ties trade patterns to factor differences between countries, and proposes that the relative abundance of factors (labour, capital etc.) shapes the production choices of a country. This model takes a genetic perspective, yet quickly becomes intractable for large numbers of factors and products, constraining detailed insights into diversification processes.

Turning to models of structural transformation and diversification, understanding these processes at a detailed level has been of keen interest for policymakers and practitioners. However, analytical intractability and measurement problems require economists to often focus on few core productive factors such as capital, labour, human capital and institutions^15,16^ and technological differences^17–19^, usually taking a genetic perspective albeit with a limited number of factors. But these models struggle to adequately describe structural transformation at a disaggregate level. Here, we exploit the fact that we can observe and measure the phenotypes, namely the presence of industries in countries, and propose a phenotypic approach to modelling the process underlying structural transformation at a detailed level.

To date, two coupled but distinct modelling approaches have emerged aiming to describe the path-dependent process of diversification based on capabilities and productive knowledge using a phenotypic approach. The first is focused on empirically estimating the number of complementary capabilities, or complexity, needed to make a product (or present in a technology or place)^4,20^. While a variety of approaches have been proposed, the foundational method to estimate product complexity^4,21^ uses information on which countries make what products to infer product capability requirements. This model assumes that complex products can only be made by countries which have many capabilities, and hence, also make many other products. It has been shown that the aggregate complexity level of a country is a strong predictor of its future income growth compared to standard variables often associated with country sophistication such as education and quality of government.

A second class of models seeks to map the path-dependent dynamical process by which countries move into new products^3^. These are connected, both theoretically and methodologically, to the study of regional and urban industrial diversification^5,7,8^, and are based on the assumption that countries will move into products similar to their current export (capability) basket. At the forefront of these models, the The Product Space^3^ is a network of products with edges based on cross-sectional export data. Under the assumption that a product pair requiring similar capabilities will be co-exported by many countries, the (cross-sectional) co-export probability of any two products is assumed to be related to the capability overlap. The location of products made by a country in this network determines its future diversification potential. Countries with products in denser parts of the network have more options, while those on the periphery share capabilities with few other products. In related work, Zaccaria et al.^22^, similarly inspired by a capability-based approach, created a taxonomy of products based on the excess conditional probability of producing a product in the presence of other products, also using the cross-sectional data. By selecting the maximum among the excess probabilities, a product hierarchy tree is generated and used to model the dynamics of the product diversification of countries. The ability of these network models, and others like it, to generate detailed metrics related to diversification processes has propelled the field into development and industrial policy-making at the global, national and regional level^2,23^.

Yet, these dual modelling approaches, capturing slightly different elements of the same underlying process, have not been unified to date. Importantly, these models are motivated via a capability-based narrative, as introduced above, but they are not underpinned by a mathematical model that explicitly takes capabilities into account. Additionally, they do not address the temporal aspect of the diversification process as a result of capability accumulation directly. Furthermore, they omit a large amount of available information on the patterns of diversification observed over the past couple of decades worldwide. Here we seek to develop a unified model, which is theoretically grounded in the path-dependent accumulation of capabilities and products, and utilises the available data for international export diversification.

Building on Hausmann and Hidalgo^24^, who developed a capability-based Leontief-like production function, we propose a model to describe the pattern of product appearances within and across countries based on capability accumulation. Within this framework, a country will jump to a new product with probability decreasing in the number of missing capabilities to make the product. We infer the capabilities possessed by a country by looking at the capabilities of the products it currently produces. The ability of a country to diversify is, hence, dependent on its current product basket. Countries with many existing products will have few missing capabilities, and many options for diversification. Hence, the pattern of product appearances contains information about the underlying capability overlap between products. We derive a relationship between the probability of a product presence (say product *i*) given the subsequent appearance of product *j*, and use this to infer the extent of capability overlap between the product pair *i* and *j*. The Ecosystem of a product *i* is then the overlap of product *i* with all other products *j*. We empirically estimate this capability overlap using product presences and appearances in international export data from 1984 to 2016.

What does it mean for a product *j* to have a large value in the Ecosystem of product *i*? There are several implications that come directly from the model. First, it means that products *i* and *j* share capabilities and the extent of overlap between the capabilities is captured by the value of the Ecosystem entry. Secondly, product *j* often precedes the product *i* in appearances in the world (if the value of the Ecosystem entry is large), giving us a directional relationship. Third, countries that have *j* have a higher probability to jump to product *i*. This dynamic aspect of the Ecosystem as captured by this precedence relationship is one of the most important differences compared to the Product Space.

In order to explore path-dependent diversification processes, we construct a weighted directed network, the Eco Space. The direction of the edges connecting nodes (products) represents export precedence, and the edge weight is given by our estimate of capability overlap. We analyse a range of network characteristics, including node in-degree and out-degree. Nodes with high in-degree (equivalent to the size of the product Ecosystem) are typically complex products, requiring many inputs. Nodes with high out-degree, on the other hand, are typically less sophisticated products which contribute to the Ecosystems of many other products. We show that the majority of non-zero directed edges (over 80%) transition from low complexity to high complexity products as we would expect under a capability-accumulation model. We also compute the node betweenness centrality, a measure of the number of shortest paths that transition through a node. Such nodes exhibit both high in- and out-degree - they are transition products typically produced by low and middle income countries as they move into more sophisticated products.

We investigate the structure of this network, finding that it exhibits a modular structure composed of a number of well-defined product communities (clusters). These communities are composed of groups of products that share similar capabilities, and are detected via an algorithm based on random walker dynamics^25^. In essence, if let jump from node to node on a network with probability proportional to edge weight, a random walker will become trapped in regions of the network exhibiting high internal connectivity. Deploying this method, we identify five stable communities in the Ecosystem network. We explore the evolution of countries based on the location of product appearances in the network: countries tend to diversify along the arrow of development starting in an origin community which is composed of high out-degree products, and eventually concentrating in a variety of distinct but interconnected destination communities composed of high in-degree products.

Finally, using an out-of-sample approach, we show that our model (empirically estimated from export data for the period 1984–2009) is informative in predicting the emergence of new products in the exports of countries for the period 2010–2016. We can interpret this result as suggesting that a country with an export basket proximate (in terms of capability gap) to a particular product is more likely to competitively export that product in the future. This model compares favourably in comparison to the Product Space^3^ in terms of the prediction of export appearances.

## Results

### Productive Ecosystems

In order to model the process of product diversification via capability accumulation, we build on Hausmann and Hidalgo^24^. According to this Leontief-based model, products require a large number of capabilities in order to be made, and countries can only make a product if they possess all the required capabilities. We denote the vector of capabilities of a product *i*, **p**_**i**_ ∈ {0, 1}^*m*^ where *m* denotes the (unknown) number of capabilities and *p*_*ik*_ = 1 if product *i* requires capability *k*. Analogously, the capability vector **c**_**n**_∈ {0, 1}^*m*^ encodes the capabilities present in country *n*. Neither of the vectors **p**_**i**_ or **c**_**n**_ are directly observable, but serve as intermediate inputs into our model.

In Hausmann and Hidalgo^24^, the authors develop a model based on the capability endowments of countries and the capability requirements of products in order to explain cross-sectional patterns in the distribution of product presences across countries. This model is based on a Leontief-like production function whereby a product *i* is produced in country *n* if and only if country *n* has all of the capabilities required by product *i*. The number of capabilities that product *i* requires is $\parallel {{\bf{p}}}_{{\bf{i}}}{\parallel }_{1}={{\bf{p}}}_{{\bf{i}}}^{T}\cdot {{\bf{p}}}_{{\bf{i}}}$ where ∥∥_1_ denotes the Euclidean 1-norm and ^*T*^ denotes the transpose of the vector. In the remainder of the paper, we only use this norm, so we will skip the subscript in the norm and the transpose sign when we are calculating inner products. Hence, country *n* produces product *i* if and only if **c**_**n**_ ⋅ **p**_**i**_ = ∥**p**_**i**_∥. The model is solved assuming that the probability that a country has/product requires a capability with a constant probability.

Focusing on modelling the temporal dynamics of diversification, and specifically product appearances, here we assume that country *n* will start making a product *i* at a future time *t*_1_, which it does not currently make, with a probability that decreases with the number of capabilities that are not present in the country but required for product *i* (at some initial time *t*_0_). Formally, if product *i* requires ∥**p**_**i**_∥ capabilities, and country *n* has **c**_**n**_ ⋅ **p**_**i**_ of them, country *n* needs to acquire ∥**p**_**i**_∥ − **c**_**n**_ ⋅ **p**_**i**_ capabilities in order to produce product *i*. We name this difference the capability gap between the capability vector of the country and capability requirement vector of the product. The probability that country *n* will start making product *i* decreases as size of this gap increases. Following Hausmann and Hidalgo^24^, we can assume that the probability of acquiring a capability is binomial with mean *q*. Hence,

1 $$P({J}_{n,i}^{{t}_{0}\to {t}_{1}}=1)={q}^{\parallel {{\bf{p}}}_{{\bf{i}}}\parallel -{{\bf{c}}}_{{\bf{n}}}\cdot {{\bf{p}}}_{{\bf{i}}}}$$

where ${J}_{n,i}^{{t}_{0}\to {t}_{1}}=1$ if product *i*, which was absent in time *t*_0_, appears in country *n* at time *t*_1_, and 0 otherwise (to minimize notational clutter, we will omit the time indices). Since 0 < *q* < 1, the probability of jump decreases with an increase in the capability gap.

We show in the ‘Methods’ section that, if we assume that the probability of a country having each capability is *w* by making a mean-field assumption, we can express the capability overlap between *i* and *j* as

2 $${E}_{i,j}={\mathrm{log}}\,\left(\frac{P({M}_{n,j}=1| {J}_{n,i}=1)}{P({M}_{n,j}=1)}\right)=-\parallel {{\bf{p}}}_{{\bf{i}}}^{{\bf{j}}}\parallel (1-w)\mathrm{log}\,(q)$$

where *M*_*n*,*i*_ = 1 if product *i* is present in country *n* at *t*_0_, and *E* is the Ecosystem matrix. The overlap vector ${{\bf{p}}}_{{\bf{i}}}^{{\bf{j}}}$ is defined as ${p}_{ik}^{j}=1$ if both *p*_*ik*_ = 1 and *p*_*jk*_ = 1 and 0 otherwise. Therefore, the probability that the product *j* is already produced in a country, given the country started making the product *i*, increases with the overlap between the capability requirements of these two products, captured by $\parallel {{\bf{p}}}_{{\bf{i}}}^{{\bf{j}}}\parallel$ up to a constant multiplicative factor.

**Fig. 1:**
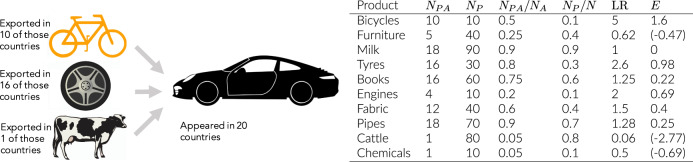
A simple toy example. This figure illustrates the main concept behind the calculation of the Ecosystem for a product. We compute the Ecosystem entries for product car. Here *N* = 100 is the total number of countries and *N*_*A*_ = 20 is the total number of appearances of cars at *t*_1_. We observe *N*_*P*_, the total number of presences of each product at *t*_0_, and *N*_*AP*_, the number of presences of the product in countries where cars appeared at *t*_1_. In this case engines, bicycles and tyres were the most over-produced in the earlier period by countries who later had an appearance of car as shown by *N*_*PA*_/*N*_*A*_. LR is the likelihood ratio of this compared to what would be expected (*N*_*P*_/*N*). Finally, $E=\mathrm{log}\,({\rm{LR}})$ contains the Ecosystem entries. Note: entries less than 0 correspond to a ratio LR less than one.

We refer to the row vector ${{\bf{E}}}_{{\bf{i}}}={\{{E}_{i,j}\}}{\,}_{j = 1,...,n}$ as the Ecosystem of a product *i*. This captures the extent of capability overlap between product *i* and each product *j*, and is calculated based on the probability that product *j* was already present when product *i* appeared. In the ‘Methods’ section, we outline how we empirically estimate the Ecosystem matrix using product presences and appearances (based on revealed comparative advantage^26^) in international trade data. Negative values are set to 0 in this matrix which corresponds to a ratio *P*(*M*_*n*,*j*_ = 1∣*J*_*n*,*i*_ = 1)/*P*(*M*_*n*,*j*_ = 1) less than 1. In order to study long term diversification trends, we create a single composite Ecosystem matrix $\widehat{{\bf{E}}}$ using data from 1984 to 2009 for 751 4-digit SITC products (in our prediction exercise, we use an out-of-sample approach to predict product appearances for the period 2010–2016). A toy example illustrating the method is shown in Fig. 1.

As discussed above, a range of approaches have been proposed to quantify the complexity of a product. Under our capability-based model, products with large Ecosystems (that have many non-zero entries in their Ecosystem vector) share capabilities with many other products. These are complex products, likely requiring a wide range of distinct capabilities. Let ${\bf{X}}=\widehat{{\bf{E}}}\,> \, 0$, an indicator matrix for the positive entries of $\widehat{{\bf{E}}}$. We define the product Ecosystem size of product *i* as the sum of row *i* of **X**, i.e., the number of non-zero Ecosystem products. We define the product Ecosystem input of product *i* as the sum of column *i* of **X**, i.e., the number of products for which product *i* is an Ecosystem product.

**Fig. 2:**
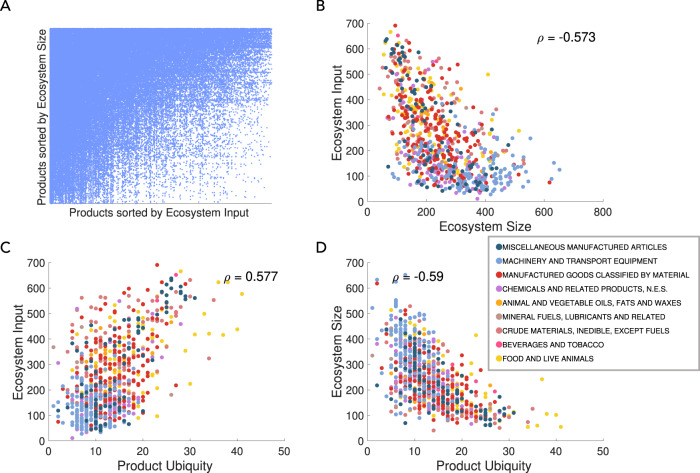
Products with a large ecosystem, which are themselves rare, are rarely inputs to the ecosystem of other products. **A** We create a single composite Ecosystem matrix using data from 1984 to 2010. Rows contain the Ecosystem entries for each product. Products in the rows are sorted by Ecosystem size, defined as the row-sum of positive entries (from high to low) and columns are sorted by Ecosystem input, defined as the column-sum of positive entries. The nested structure of the matrix shows that large size Ecosystems products rely on capabilities present in both small and large Ecosystem input products (top rows are densely filled), whereas small Ecosystem products rely on capabilities present in products common to many Ecosystems (bottom rows are filled only on the left hand side). **B** We plot the Ecosystem size vs. Ecosystem input. The negative relationship confirms that products with a large Ecosystem are rarely inputs to the Ecosystems of other products. **C**, **D** We show that the Ecosystem input (size) is positively (negatively) correlated with product ubiquity.

Figure 2 A presents a visual representation of the entries in matrix **X**, where blue dots in row *i* correspond to non-zero entries in the Ecosystem vector of product *i*. Products are sorted by Ecosystem size in the rows and Ecosystem input for the columns. The nested structure of the matrix shows that large size Ecosystems products rely on capabilities present in both small and large Ecosystem input products (top rows are densely filled), whereas small Ecosystem products rely on capabilities present in products common to many Ecosystems (bottom rows are filled only on the left hand side). These patterns are consistent with the nested pattern observed in cross-sectional data for product presences by Bustos et al.^21^.

Consistent with our capability-based model, Fig. 2B shows that almost no products have both a large Ecosystem size and input (i.e. the top right corner is empty). In other words, as we would expect, products requiring many capabilities with a large Ecosystem size are not simultaneously (input) Ecosystem products for many products. Machinery and transport equipment products (blue) tend not to be high input products, while most food products (yellow) have a small Ecosystem size.

Next, we explore how the Ecosystem input and Ecosystem size relate to product ubiquity, which is the number of countries that has comparative advantage in the product (above the threshold). According to Hidalgo and Hausmann^4^, high product ubiquity is associated with low complexity products as these products can be made by many countries. Figure 2C shows the positive relationship between Ecosystem input and product ubiquity. Note that the denominator of the Ecosystem equation, Eq. (2), is effectively equal to the product ubiquity. For this reason one might be concerned that high ubiquity products may not be present in the Ecosystems of many products. Figure 2C shows that this is not the case. Figure 2D confirms the negative relationship between Ecosystem size and product ubiquity.

Table 1 shows the top 15 products in terms of Ecosystem size, and the top 15 products in terms of Ecosystem input. In the first case we observe a range of sophisticated products, including machinery and electrical appliances, vehicles and engines, and chemicals. In the second case we have less complex products, including raw textiles and fabrics, simple garments and basic chemicals. In Supplementary Note 1 of the Supplementary Information, we show that the overall pattern of entries in the Ecosystem matrix is robust to alternative Revealed Comparative Advantage (RCA) thresholds associated with product absences, presences and appearances, and to variation in the time period used.

**Table 1:** Largest Ecosystem size and input products.

	(1)	(2)
Rank	Largest Ecosystem size products	Largest Ecosystem input products
1	Complete digital central processing units, digital processors	Asbestos
2	Diodes, transistors, photocells, etc.	Cotton fabrics, woven, unbleached, not mercerized
3	Mechanically propelled railway, tramway, trolleys, etc.	Drawn or blown glass (flashed glass), unworked, in rectangles
4	Rail locomotives, electric	Kelem, schumacks and karamanie rugs and the like
5	Railway, tramway passenger coaches, etc, not mechanically propelled	Sisal, agave fibres, raw or processed but not spun, and waste
6	Domestic dishwashing machines	Carpets, carpeting and rugs, knotted
7	Uranium depleted in U235, thorium, and alloys, nes, waste and scrap	Raw cotton, excluding linters, not carded or combed
8	Power hand tools, pneumatic or non-electric, and parts thereof, nes	Mineral or chemical fertilizer, potassic
9	Chemical wood pulp, soda or sulphate	Wool greasy or fleece-washed of sheep or lambs
10	Peripheral units, including control and adapting units	Linens and furnishing articles of textile, not knitted or crocheted
11	Motor vehicles piston engines, headings: 722, 78, 74411 and 95101	Silk worm cocoons and silk waste
12	Complete digital data processing machines	Jute, other textile bast fibres, nes, raw, processed but not spun
13	Other sound recording and reproducer, nes, video recorders	Blouses
14	Silicones	Other outer garments
15	Office machines, nes	Fur clothing (not headgear) and other articles made of furskins

Column (1) shows the top 15 products in terms of Ecosystem size and column (2) shows the top 15 products in terms of Ecosystem input. In the first column we observe a range of sophisticated products including engines, chemicals, equipment and vehicles. In the second column, overall we have less complex products including food, textiles, metals and basic chemicals.

### The arrow of development

Countries diversify into new products that are similar (in terms of required capabilities) to what they currently produce. In order to model this process, we construct a network of products. Directed edges connect the products: there is an arrow from node *j* to node *i* if product *j* is in the Ecosystem of product *i*, which implies that *j* tends to be produced before *i* appears. The weight of the edge is an estimate of capability overlap between *i* and *j* as determined by the corresponding positive Ecosystem entry.

We can ask questions such as: do we observe clusters of products sharing many capabilities? Which products are most likely to be part of a development path? How do countries diversify in this network?

Formally, the Eco Space is a network with *n* nodes (or vertices). The structure of any network can be encoded by the adjacency matrix ${\bf{A}}\in {{\mathcal{R}}}^{n\times n}$ where entries *A*_*ij*_ correspond to the weight of the directed edge from node *i* to node *j*. In this case, ${{\bf{A}}={({\bf{X}}\circ \widehat{{\bf{E}}})}^{\text{T}}}$ is the adjacency matrix for the Eco Space where ^T^ denotes the transpose of the matrix and ∘ denotes the Hadamard product (element-wise multiplication) operator. Using this adjacency matrix we can compute a host of network metrics, including, for example, the in-degree *d*_*i*_ = ∑_*j*_ *X*_*ji*_ (equivalent to the Ecosystem size), and the in-strength, *s*_*i*_ = ∑_*j*_ *A*_*ji*_ for each node *i*. Note that the in-degree is exactly Ecosystem size shown Fig. 2. Similarly, the node out-degree is the Ecosystem input.

**Fig. 3:**
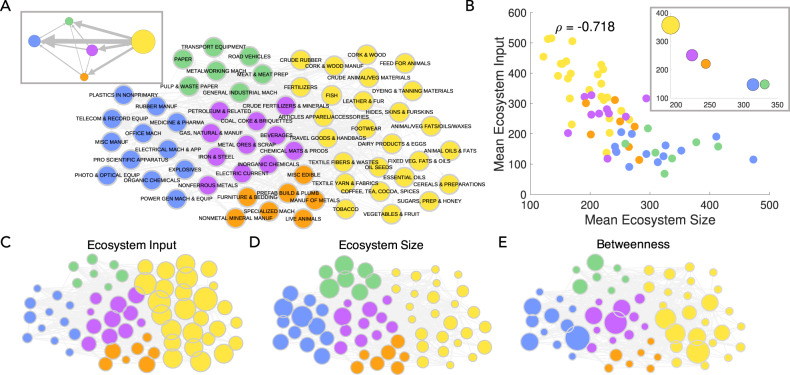
The arrow of development can be described by the Ecosystem network. **A** We construct a reduced representation of our Ecosystem network at a 2-digit product division level (63 divisions). The edges are the mean edge weights between 2-digit node groupings in the 4-digit product network (not shown). Nodes are coloured by node community. In the inset, we show the overall network aggregated to the community level. Node size in the inset corresponds to the number of 2-digit products within each community. **B** The relationship between mean Ecosystem input and mean Ecosystem size at the 2-digit division level. In the inset, we show the same relationship at the community level with node size corresponding to the number of 2-digit product divisions in the community. **C**, **D** Ecosystem network at 2-digit level with node sizes proportional to the mean Ecosystem size and mean Ecosystem input. We can clearly see that the large Ecosystem input products are located on the right and large Ecosystem size products are located in the left. Hence, the arrow of development often follows this right to left trajectory. **E** We compute the betweenness centrality of each node (in the 4-digit network), a measure of the number of times a shortest path between any two nodes traverses the node, and visualise the mean for each 2-digit division. These transition products have high in and out degree.

We construct a reduced version of this network, calculating the mean edge weight between products in 2-digit divisions (there are 63 2-digit product divisions). Figure 3A illustrates the directional relationship between divisions, showing mean edge weights over a threshold of 0.2 (this includes about the top 12.5% of edges). The software programme Gephi^27^ has been used to generate the network layout using the automated Force Atlas algorithm.

In order to probe the structure of this network, we search for clusters of nodes (communities) which exhibit high internal connectivity, but sparse connections between communities. Within this context, communities represent groups of products with shared capabilities. The presence of modular structure, whereby sparse connections lie between clusters, could prove an obstacle to diversification processes, as countries become trapped in a community. This type of network topology has been detected in a wide range of networks, particularly social and biological networks, and is often indicative of an underlying functional organisation^28,29^.

There are a large variety of approaches to community detection, many based on comparison of the network structure to a statistical null model (i.e., the connectivity structure if edges were placed at random, see Fortunato^28^ for a review). Most traditional methods seek to find a single optimal partition, yet this approach often neglects the presence of modular structure at a range of scales (e.g. few large communities vs many small communities). Here we apply the Stability algorithm^25^, which is based on the dynamics of a random walker on a network. In essence, a random walker jumps from node to node with probability proportional to edge weight. If the walker gets trapped in a region of high connectivity, the corresponding group of nodes corresponds to a tightly knit community. The longer the walker jumps, the larger the communities she finds. Hence, a time parameter enables us to control the scale (from many small communities to few larger communities) at which communities are uncovered. In the Supplementary Information, we describe the optimization process to find a node partition for which the algorithm is most robust.

We apply this algorithm to our reduced two-digit network representation. In Fig. 3A, we observe clear groupings, with food, animals, crude materials and textiles dominating the yellow community on the right-hand-side. As we move to the left we observe clusters of transportation equipment and machinery (green), and manufacturing (orange). In the center (purple) we have processed petroleum products, metals and chemicals/plastics. Moving to the far left (blue), we have sophisticated products such as medicines and pharmaceuticals, scientific equipment and electrical machinery. The inset shows a further reduced version of the network, where each node corresponds to a community. We clearly observe the arrow of development, as countries begin their development path in the yellow community, and progressively jump into new products located in the center and far left of the network layout. The blue and green (and to a lesser extent orange and purple) communities represent destination products typically produced in highly developed nations.

Figure 3B shows the relationship between mean Ecosystem input and mean Ecosystem size at the 2-digit division level (63 divisions), with points coloured by community assignment. In the inset, we show the same relationship aggregated to the community level. We confirm that the yellow community is dominated by high Ecosystem input but low Ecosystem size products. On the other hand, the blue and green communities are dominated by high Ecosystem size but low Ecosystem input products. Figure 3C, D shows the Ecosystem network at 2-digit level with node sizes proportional to the mean Ecosystem size and mean Ecosystem input. We can clearly see that the large Ecosystem input products are located in the right and large Ecosystem size products are located in the left.

We can also extract information about intermediate steps. We compute the betweenness centrality of each node, a measure of the number of times a shortest path between any two nodes traverses the node. In Fig. 3E we visualise the mean betweenness for each 2-digit division. These transition products tend to have high in and out degree - they are stepping stones.

**Fig. 4:**
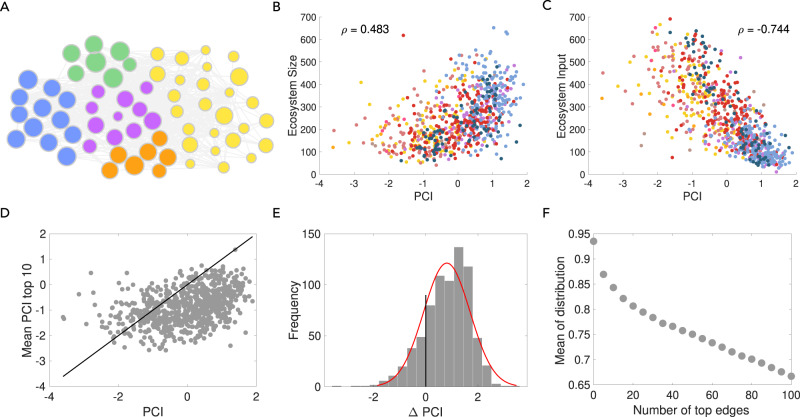
Large Ecosystem products tend to be complex products with high product complexity index (PCI). **A** Here we show the mean PCI of products in 2-digit division. **B**, **C** Large Ecosystem sizes are associated with complex products, and less complex products are inputs to many Ecosystems. Colour legend is the same as Fig. 2. **D** The relationship between the PCI of a product and the mean PCI of its top 10 incoming neighbours. 83% of the products are to the right of the 45 degree line, showing that non-zero directed Ecosystem edges go from low-complexity to high-complexity products. **E** For each product, we compute the PCI of the product minus the mean PCI of its top *x* = 10 incoming neighbours. The histogram shows a clear bias towards positive values—the PCI of the product is higher than its incoming neighbours. **F** By looking at the mean of the distribution across a range of *x*, we find that, as expected, this mean moves towards zero as we increase the number of neighbours.

An alternative widely-used metric to estimate the productive sophistication of a product, the product complexity index (PCI)^4^, is derived from export data based on the hypothesis that rare or complex products are only made by few countries that possess many capabilities (and, therefore, produce many other products). As shown by Hausmann et al.^2^, higher complexity products are mostly associated with (rare) highly diversified developed countries (who produce both common and rare products) while lower complexity products are produced in countries at all levels of development. Here, we investigate the relationship between the Ecosystem of a product and its PCI value. We would expect that products with a high PCI value, requiring many (and rare) capabilities, have a large Ecosystem size. On the other hand, products with a low PCI value, needing fewer more common capabilities, would be expected to have few Ecosystem products. Figure 4A shows the distribution of PCI within the Eco Space (e.g. the mean PCI of products within the 2-digit divisions). As confirmed in Fig. 4B, high PCI products coincide with those with a high Ecosystem size. On the other hand, Fig. 4C shows that low PCI products tend to be inputs to the Ecosystem of many products.

If capability accumulation underlies the development process, we expect countries to move from less complex products towards sophisticated products over time. Hence, we expect diversification from low complexity to high complexity products as countries upgrade their complexity level. We look at the directed edges between products of different complexity levels and ask, is it more likely that an edge connects a lower complexity node to a higher complexity node? In other words, are the input products within a product’s Ecosystem less complex than the product itself? Hence, we are interested to see whether the directionality of edges moves from lower to higher PCI products. For each node we show the relationship between its own PCI, and the mean PCI of its top *x* = 10 incoming neighbours (Ecosystem entries) in Fig. 4D. We observe that most products (83% of products) have a higher PCI than the mean of their top 10 Ecosystem products. Next, we compute the PCI of the product minus the mean PCI of its top *x* = 10 incoming neighbours. The histogram in Fig. 4E shows a clear bias towards positive values—the PCI of the product is higher than its incoming neighbours. Finally, by looking at the mean of this distribution across a range of *x* in Fig. 4F, we find, as expected, that the mean decreases as we increase the number of neighbours.

**Fig. 5:**
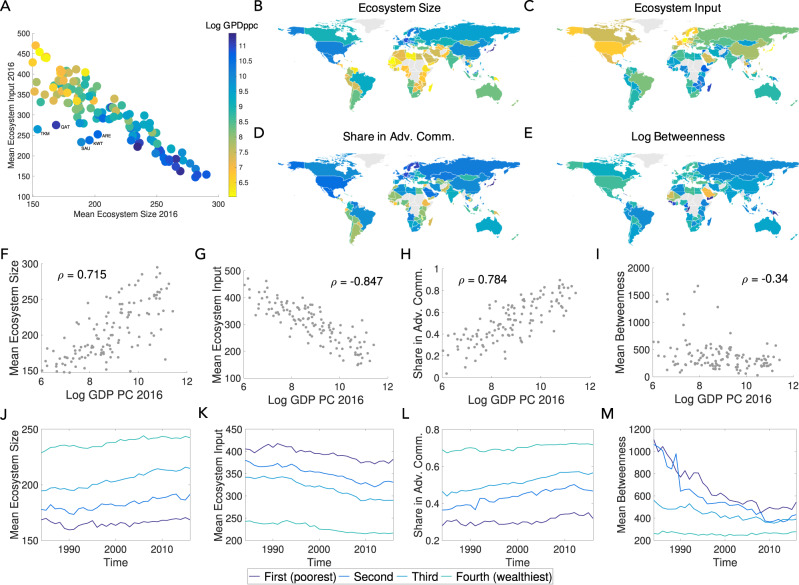
The poorest countries dominate products with large Ecosystem input, while wealthy countries tend of have products with large Ecosystem size. **A** First, we show the mean Ecosystem size vs. mean Ecosystem input level for each country. The points are coloured according to per capita GDP. There is a clear negative relationship—countries that tend to have many large Ecosystem size products also seem to have few high Ecosystem input products and vice versa. We label the outliers, which are mostly oil or natural gas rich countries. **B**–**E** We show maps with countries shaded by mean Ecosystem size, Ecosystem input, share of products in advanced communities and the log-betweenness centrality of their products for year 2016. The colormap is rescaled for each variable, with yellow corresponding to minimum value and dark blue corresponding to maximum value. **F**–**I** The poorest countries dominate products with large Ecosystem input, and transition products with high betweenness centrality. On the other hand, wealthy countries tend to have the largest Ecosystem size, and have a higher share of products in advanced communities (all data for 2016). **J**–**M** Over time, from 1984 to 2016, we observe that poor countries moved out of transition products with high betweenness centrality, and middle and high income countries increased their share of products in the advanced communities.

How do these product attributes relate to the wealth of the countries who produce them? For each country, we compute the mean Ecosystem size and mean Ecosystem input level of the products it exports with RCA higher than the presence threshold. Figure 5A plots these values for each country, where the points are coloured according to GDP per capita. There is a clear negative relationship between the size and the input, with higher GDPpc countries—located in the lower right portion of the graph—exhibiting mainly high Ecosystem size/low Ecosystem input products. We label the outliers in the graph, which are mostly oil or natural gas rich countries. Figure 5B, C shows maps with countries shaded by mean Ecosystem size and Ecosystem input. Finally, Fig. 5F, G confirms that wealthier countries export products with high mean Ecosystem size and low mean Ecosystem input.

We can also examine the share of products in advanced communities (defined here as all communities except the yellow community), and the log-betweenness centrality of their products, for each country. Figure 5D, H confirm that wealthier countries tend to be concentrated in advanced communities. Products with high betweenness centrality can be seen as transition products, and would be expected to be produced by low-middle income countries as seen in Fig. 5E, I.

Next, we explore the evolution of these metrics over time (1984–2016), dividing countries into four equally-sized income groups (by GDP per capita). Figure 5J–M show that middle income countries increased their share of products in high Ecosystem size products (and those in advanced communities), while both poor and middle income countries decreased their share of products in high Ecosystem input products, and moved out of transition products with high betweenness centrality.

**Fig. 6:**
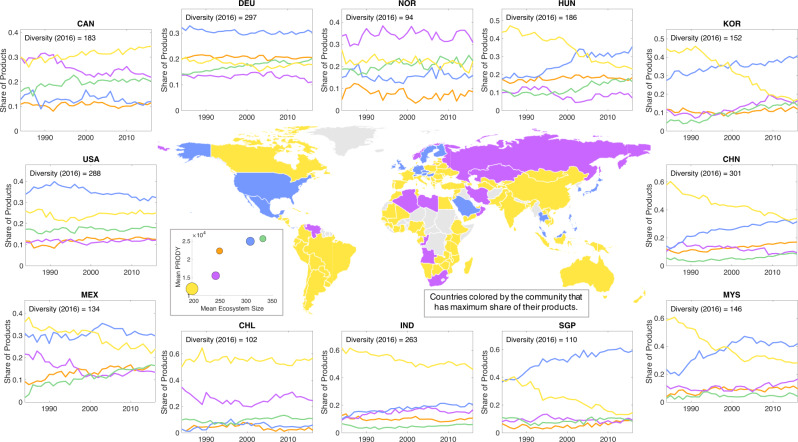
Structural transformation through the lens of Ecosystem communities. (Center) Countries are coloured according to the community that has the highest share of their products. (Center inset) In the inset we show the mean Ecosystem size vs. PRODY^30^ for the products in each community. PRODY captures the wealth associated with a product and is computed as the RCA weighted average of the GDPpc of the countries in which the product is present. (Outer figures) For a variety of countries, we show the evolution of each country’s share of products in each of the communities over time, from 1984 to 2016. We can distinguish between those who transitioned early in the period in the 1980s (SGP-Singapore), those who transitioned in the middle of the period around the year 2000 (HUN-Hungary, KOR-Korea, MYS-Malaysia, and MEX-Mexico) and those who transitioned more recently (CHN-China).

How do individual countries transform their export composition over the communities we identified from the Ecosystem network? Here, we explore in more detail the temporal evolution of the product basket of nations as they diversify into new products and move through the network over time. Using data for 2016, the central map in Fig. 6 shows countries shaded by the colour of community that has the highest share among their products. We observe that a majority of countries currently export products concentrated within three communities: yellow (food, animals, crude materials and textiles), purple (petroleum products, metals and chemicals/plastics), and blue (medicines and pharmaceuticals, scientific equipment and electrical machinery). In Supplementary Note 5 of Supplementary Information, we show the relative share of each country’s export presences across each individual community. The inset next to the map shows the mean Ecosystem size vs. PRODY^30^, which is calculated as the RCA weighted average of the GDPpc of countries for each product. The size of the points is the number of 2-digit products in the community. This plot confirms that the yellow and purple communities are dominated by products exported by low GDPpc nations, while the blue community is dominated by products exported by high GDPpc nations.

Over time, from 1984 to 2016, we see that many countries go through transformations by changing their share of products in different communities. For example, we observe a number of countries transitioning over this period from a concentration of products in the yellow community to those in the blue community. We can distinguish between those who transitioned early in the period in the 1980s (SGP-Singapore), those who transitioned in the middle of the period around the year 2000 (HUN-Hungary, KOR-Korea, MYS-Malaysia, and MEX-Mexico) and those who transitioned more recently (CHN-China). India (IND) appears to be on this path, with a future transition on the horizon. Norway (NOR) is dominated by products in the purple community, while Germany (DEU) and the USA are dominated by products in the blue community.

### Predicting product appearances

Beyond analysing network properties and diversification paths, we wish to assess whether the model is informative in predicting the appearance of new products, or equivalently the export of new products with comparative advantage, for the set of all countries. For each product-country pair, this translates to estimating the exponent in Eq. (1), the gap between the capabilities required by the product and the capabilities held by the country. Our strategy is to infer the capabilities required for a product by looking at its maximum Ecosystem entry, which is an estimate of the maximum capability overlap with all other products. While we simply introduce our new metric here, a comprehensive derivation and explanation is provided in the Methods Section.

To predict the likelihood of an appearance of product *i* in country *c*, we estimate the capability gap in the exponent of Eq. (1) via

3 $${d}_{n,i}^{E}={q}^{\mathop{\max }\nolimits_{j}{\hat{E}}_{i,j}-\mathop{\max }\nolimits_{j\in {{\mathcal{J}}}_{n}}{\hat{E}}_{i,j}}$$

where ${{\mathcal{J}}}_{n}$ is the set of products present in country *n*. We call this metric the Ecosystem density. We complement our derivation in the ‘Methods’ section with a graphical explanation. In order to reduce noise, we take the mean value over the top *k* = 25 entries for each $\mathop{\max }\nolimits_{j}{\hat{E}}_{i,j}$ and $\mathop{\max }\nolimits_{j\in {{\mathcal{J}}}_{n}}{\hat{E}}_{i,j}$. The robustness of our results in terms of parameter *k* is given in Supplementary Note 3.3 of the Supplementary Information. We note that the Ecosystem encoded in matrix $\hat{E}$ was constructed using data from 1984 to 2009. Product presences in Eq. (3) are measured in 2010, and we seek to predict appearances during the period 2010–2016.

We measure the predictive power of our variables using area under the curve (AUC) of the receiver operating characteristic, which plots the rate of true positives of a continuous prediction criterion as a function of the rate of false positives. For a standard probit model, Table 2 shows that Ecosystem density variable has predictive power for country-product appearances with AUC = 0.715 (column 1), increasing to AUC = 0.813 when country and product fixed effects are included (column 4).

**Table 2:** We seek to predict appearances during the period 2010–2016.

	(1)	(2)	(3)	(4)	(5)	(6)	(7)
Variables							
Eco Density	9.4924*** (0.3934)	11.3625*** (0.5415)	9.0869*** (0.4314)	10.5294*** (0.7503)		10.4047*** (0.4731)	9.4294*** (0.7246)
PS Density					1.7168*** (0.0986)	−0.6110*** (0.1451)	6.4706*** (0.6467)
Constant	−10.4983*** (0.3650)	−12.1721*** (0.5118)	−9.7782*** (0.4467)	−11.1787*** (0.7240)	−1.9996*** (0.0167)	−11.2588*** (0.4285)	−10.3521*** (0.6941)
Observations	49,352	49,195	39,420	39,280	49,352	49,352	39,280
Number of App.	1831	1831	1831	1831	1831	1831	1831
Country FE	No	Yes	No	Yes	No	No	Yes
Product FE	No	No	Yes	Yes	No	No	Yes
Pseudo *R*^2^	0.065	0.112	0.114	0.164	0.015	0.066	0.172
AUC	0.715	0.773	0.764	0.813	0.623	0.718	0.819

For a standard probit model, we see that the Ecosystem density metric has predictive power for country-product appearances with AUC=0.715 (column 1), increasing to AUC=0.813 when country and product fixed effects are included (column 4), and compares favourably to a similar metric based on the Product Space (column 5). The number of observations in columns 3, 4 and 6 are lower because of the inclusion of the country and product fixed effects. With country fixed effects, the countries that do not observe any jumps drop out. With product fixed affects, the products into which no countries jump drop out.

Robust standard errors in parentheses. ***p < 0.01, **p < 0.05, *p < 0.1.

We compare the ability of this metric to predict product appearances with the Product Space density^3,31^, a predictive metric based on the structure of the Product Space (see ‘Methods’ for details). We find that our Ecosystem based metric outperforms the Product Space density which has AUC = 0.623 (column 5, no fixed effects). When both measures are included together, the sign of the Product Space density becomes negative after controlling for the Ecosystem density in Column 6, but it recovers its positive sign when the fixed effects are included in Column 7. An increase in the predictive power is also evident in the pseudo-R^2^ measure, which increases to 6.5% from 1.5% when Ecosystem density is used compared to the Product Space density.

**Fig. 7:**
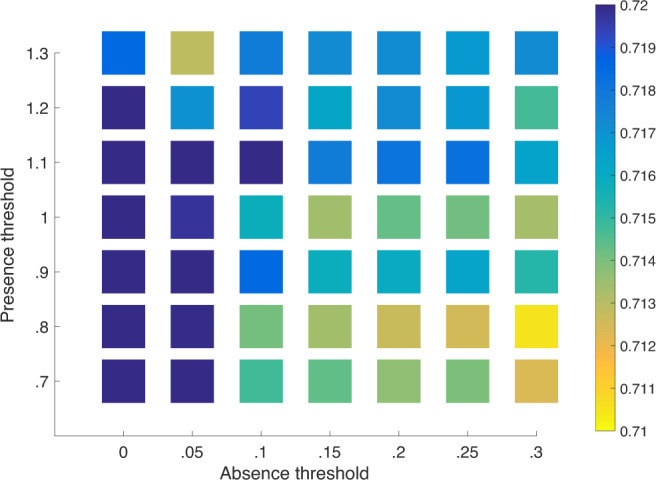
Heat map for the AUC values for various combinations of parameters *τ*_0_ (absences) and *τ*_1_ (presences). The reported AUC values correspond to an equivalent regression to column 1 of Table 2 with presence and absence thresholds reported in row and column labels, respectively.

Product appearances are dependent on two thresholds: one for product absences (*τ*_0_) and one for product presences (*τ*_1_), see Eqs. (6) and (7) in the ‘Methods’ section. The default values of these, discussed below, are *τ*_0_ = 0.1 and *τ*_1_ = 1. As we decrease *τ*_0_, we have fewer absences (and hence fewer possible appearances). As we increase *τ*_1_, we also have fewer appearances. In order to explore variation in the predictive ability of our model for variation in these parameters, in Fig. 7, we show a heat map for the AUC values for various combinations of *τ*_0_ and *τ*_1_ corresponding to column 1 of the table. We observe, for reasonable combinations *τ*_0_ and *τ*_1_, the base-line (no fixed effects) AUC scores are consistently close to 0.72.

In the Supplementary Information, we apply a number of tests to assess the robustness of our results:

- We vary the number of products used in the computation of the maximum in the exponent of *q* that we use to create our density measure in Eq. (3), further explained in the ‘Methods’ section (Supplementary Fig. 10).
- We split the countries into different categories such as high vs. low per capita GDP, high vs. low complexity and high vs. low export volume (Supplementary Table 1).
- In order to test for redundancy in the product classification, we omit products from the same SITC division in the construction of the Ecosystem (columns 1 and 2 of Supplementary Table 2).
- We split the products into different groups such as manufacturing vs. non-manufacturing, high vs. low complexity, high vs. low ubiquity, and high vs. low export volume (columns 3–10 of Supplementary Table 2).
- We modify our criteria in order to observe a jump of a country into a new product in terms of the number of years of product absence followed by product presence required (Supplementary Tables 3–5).
- We use an alternative measure of RCA, which compares a country’s per capita production levels in a product to the world’s overall per capita production of the product to reveal the comparative advantage (Supplementary Table 6).

Our results remain robust to these various tests.

Our regression results indicate our Ecosystem measure captures path-dependent diversification patterns and surpasses the current best comparator, the Product Space, in its predictive ability. It is important to acknowledge that our results do not predict future jumps perfectly. Our Ecosystem density measure captures potential products which require few additional capabilities for countries to move into, but given the limited resources of countries to exploit these adjacent products, not all possible jumps are realised. As a consequence, we are trying to predict rare events, only 1831 jumps were observed out of 49,352 absences, which is close to rate of 3.7%. In addition, there are many other factors that prompt countries to begin production of new products for export, including path-defying factors^32,33^ which are not captured by our model.

## Discussion

Classical growth and trade theory has struggled to reconcile macro variables such as factor endowments with differences in the productive structure and know-how of nations. One approach would be to increase the number of factors measured and write down more detailed production functions to understand the dynamics. A complementary approach might take an agnostic stance towards the identity of the capabilities or factors but focus on the development paths associated with this deeply granular process. In this paper, we took the latter approach and, inspired by early approaches to the study of genetics, we develop a model for product diversification based on capability accumulation.

We propose a new metric, the Ecosystem of a product, which contains information on other products sharing a high-level of capability overlap. Empirically, this is the set of pre-existing products that are typically necessary for a future appearance of that product. Given the temporal nature of this measure, we construct a directed network, the Eco Space, to describe probable development paths. Exploiting tools from network science, we identify product clusters and transition sectors governing dynamics on the network. Finally, we show that the model is a good predictor of export diversification, performing favourably compared to the well-known Product Space framework^3^.

This work contributes to both the theoretical literature on the modelling of capabilities and knowledge accumulation, and more generally the processes underlying economic growth. It is particularly relevant for the literature on economic complexity^4^, and the on-going search for empirical methods to quantify, measure and validate complexity^3,20,34,35^. Similarly, it is embedded in the literature on path-dependent diversification^3,5,7^, including regional dynamics and related varieties similarly derived from an evolutionary or capability-based perspective. Future work could include estimating this model for industry employment or establishment data, which provides additional information on domestic production (and by extension domestic and service capabilities) not contained in export data^31,36^.

Our framework can also be potentially applied to a range of other settings where path-dependent diversification occurs. The first obvious extension is to the regional or urban setting where firms/industries need specific locally available capabilities to flourish. This will result in a path-dependent process of diversification, which underlies some of the dynamics behind industrial cluster formation^37^ and urban agglomeration^8^. In a similar vein, technology adoptions by countries^38^ also follow a path-dependent process: many technologies require other technologies to be present in advance in a country. Finally, in biology, from where we borrow the term ecosystem, organisms require the presence of other animals or plants to populate a location, and, hence, this mechanism also leads to path-dependent dynamics. This process is intimately linked to observed nested structure emphasised in the ecology (and economics) literature^21,39–41^.

As confidence in market efficiency has declined, particularly since the 2008-9 financial crisis, industrial policy has enjoyed somewhat of a global resurgence^42^. Although, there have been clear examples of path-defying changes^32,33^, the metrics derived here aim to aid countries or regions to connect their current productive capabilities to future possibilities. In particular, we hope that the Ecosystem metric is helpful to policymakers seeking to analyse the preparedness of a nation or region to move into a new product, or trying to identify key transition sectors which could open up future opportunities. Additionally, policymakers can also use this methodology retrospectively to identify market failures. This is possible by identifying products that had a high likelihood of appearance, but have not yet been observed. Factors that prevented the appearance of these products can then be investigated. While there are clear policy applications for our work, it is also prudent to highlight limitations of the model. Firstly, although an improvement on previous approaches, the predictive power of the model suffers from false positives since many possible jumps are not realised due to external factors. Secondly, evolving production technologies impact the underlying capability requirements of many products, leading to an evolution of Ecosystem matrix over time, albeit at a slow pace. Based on this, and the predictive power of our model over a five-year period, we suggest that this tool is most suited to deliver short to medium term policy insights. Overall, we believe this methodology will be a valuable asset for policymakers.

## Methods

### The model

Let *M*_*n*,*i*_ = 1 if product *i* is present in country *n*, and otherwise 0. Similarly, let *J*_*n*,*i*_ = 1 if product *i* appeared in country *n*, and otherwise 0.

For a product *i* and a country *n*, **p**_**i**_ ∈ {0, 1}^*m*^ is the capability requirement vector of product *i*, and **c**_**n**_ ∈ {0, 1}^*m*^ represents the capabilities present in country *n* with *m* representing the number of the capabilities. Following Hausmann and Hidalgo^24^, country *n* makes the product *i* if country *n* has all necessary capabilities to make *i*. Formally:

$${M}_{n,i}=1\ \iff \ \parallel {{\bf{p}}}_{{\bf{i}}}\parallel ={{{\bf{c}}}_{{\bf{n}}}}^{\text{T}}.{{\bf{p}}}_{{\bf{i}}}.$$

where ∥∥_1_ denotes the Euclidean 1-norm and ^T^ denotes the transpose. We drop the subscript of the norm and transpose sign for notational brevity. We assume that the country will jump to the product upon acquisition of all missing capabilities required to make the product. Hence, the probability of a jump depends on the capability gap between the country and the product capability vectors:

$${{{\Delta }}}_{n,i}=\parallel {{\bf{p}}}_{{\bf{i}}}\parallel -{{\bf{c}}}_{{\bf{n}}}.{{\bf{p}}}_{{\bf{i}}}.$$

We wish to quantify the likelihood of country *n* producing product *i* given that the country is already producing product *j*. We can split the capability vector of product *i* into two parts, one which contains the capabilities overlapping with *j*, and other the non-overlapping capabilities. We write ${{\bf{p}}}_{{\bf{i}}}={{\bf{p}}}_{{\bf{i}}}^{{\bf{j}}}+{\overline{{\bf{p}}}}_{{\bf{i}}}^{{\bf{j}}}$, where

- ${p}_{ik}^{j}=1$ if both *p*_*ik*_ = 1 and *p*_*jk*_ = 1 and 0 otherwise, and
- ${\bar{p}}_{ik}^{j}=1$ if *p*_*ik*_ = 1 and *p*_*jk*_ = 0 and 0 otherwise.

Since country *n* is already making product *j*, it has all the necessary capabilities for it. Hence, the probability that country *n* starts making product *i* can be expressed as:

$$P({J}_{n,i}=1| {M}_{n,j}=1)={q}^{\parallel {\overline{{\bf{p}}}}_{{\bf{i}}}^{{\bf{j}}}\parallel -{{\bf{c}}}_{{\bf{n}}}\cdot {\overline{{\bf{p}}}}_{{\bf{i}}}^{{\bf{j}}}}$$

where *q* is the mean probability of acquiring a capability under a binomial model. We can apply Bayes’ Rule:

$$\begin{array}{lll}P({M}_{n,j}=1| {J}_{n,i}=1)&=&\frac{P({J}_{n,i}=1| {M}_{n,j}=1)P({M}_{n,j}=1)}{P({J}_{n,i}=1)}\\ &=&\frac{{q}^{\parallel {\overline{{\bf{p}}}}_{{\bf{i}}}^{{\bf{j}}}\parallel -{{\bf{c}}}_{{\bf{n}}}\cdot {\overline{{\bf{p}}}}_{{\bf{i}}}^{{\bf{j}}}}}{{q}^{\parallel {{\bf{p}}}_{{\bf{i}}}\parallel -{{\bf{c}}}_{{\bf{n}}}\cdot {{\bf{p}}}_{{\bf{i}}}}}P({M}_{n,j}=1)\\ &=&{q}^{-(\parallel {{\bf{p}}}_{{\bf{i}}}^{{\bf{j}}}\parallel -{{\bf{c}}}_{{\bf{n}}}\cdot {{\bf{p}}}_{{\bf{i}}}^{{\bf{j}}})}P({M}_{n,j}=1)\end{array}$$

and take logarithms:

$${\mathrm{log}}\,\left(\frac{P({M}_{n,j}=1| {J}_{n,i}=1)}{P({M}_{n,j}=1)}\right)=(\parallel {{\bf{p}}}_{{\bf{i}}}^{{\bf{j}}}\parallel -{{\bf{c}}}_{{\bf{n}}}\cdot {{\bf{p}}}_{{\bf{i}}}^{{\bf{j}}}){\mathrm{log}}\,(1/q).$$

If we assume that the probability of a country having each capability is *w*, this expression becomes

4 $${E}_{i,j}={\mathrm{log}}\,\left(\frac{P({M}_{n,j}=1| {J}_{n,i}=1)}{P({M}_{n,j}=1)}\right)=-\parallel {{\bf{p}}}_{{\bf{i}}}^{{\bf{j}}}\parallel (1-w){\mathrm{log}}\,(q).$$

Hence, the probability that the product *j* is already produced in a country, given the country started making the product *i*, increases with the overlap between the capability requirements of these two products, captured by $\parallel {{\bf{p}}}_{{\bf{i}}}^{{\bf{j}}}\parallel$ up to a constant multiplicative factor.

### Algorithm

We construct the Ecosystem matrix $\hat{E}$ using export data from the Standard International Trade Classification (SITC) revision 2 at the 4-digit level beginning in 1984 using data from Harvard Dataverse present at https://dataverse.harvard.edu/dataverse/atlascleaned by Bustos and Yildirim.

In order to estimate the matrices *M* and *J*, we measure product presences and appearances via international export competitiveness. In particular, we measure the intensity with which a country exports each product by computing its Revealed Comparative Advantage (RCA), first proposed by Balassa^26^. The RCA that a country has in a product is defined as the ratio between the share of the product in the country’s export basket and the overall share of the product in the global export basket. Equivalently, we can also think of RCA as the share of the country in the product divided by the total share of the country in the world exports. A product is over-represented in a country’s export basket if its RCA is above a threshold.

Formally, if *X*_*n*,*i*_ is equal to the export of country *n* in product *i*, then the RCA of country *n* in product *i* is defined as:

5 $${R}_{n,i}=\frac{{X}_{n,i}/{\sum }_{k}{\text{X}}_{k,i}}{{\sum }_{i}{X}_{n,i}/{\sum }_{k,i}{\text{X}}_{k,i}}$$

6 $${M}_{n,i}=\left\{\begin{array}{ll}1&\,{\text{if}}\,\ {R}_{n,i}\,> \,{\tau }_{1}\,{\text{in}}\,t\\ 0&\,\text{otherwise}\,\hfill\\ \end{array}\right.$$

An appearance of product *i* in country *n* is defined as:

7 $$P({J}_{n,i}^{{t}_{0}\to {t}_{1}}=1)=\left\{\begin{array}{ll}1&{\text{if}}\,\ {R}_{n,i}\,<\,{\tau }_{0}\,{\text{in}}\,{t}_{0}\,{\text{and}}\,{R}_{n,i}\,> \,{\tau }_{1}\,{\text{in}}\,\ {t}_{1}\\ 0&\text{otherwise}\hfill\,\end{array}\right.$$

Since we will aggregate all jumps for each country-product pair in the analysis below, we will drop the time indices in the *J* matrix.

Based on our definitions of jumps and presences, we compute the entries ${\hat{E}}_{i,j}$ as follows:

8 $${\hat{E}}_{i,j}={\mathrm{log}}\,\left(\right.P({M}_{n,j}=1| {J}_{n,i}=1)/P({M}_{n,j}=1)\left)\right..$$

We will show how we build the *P*(*M*_*n*,*j*_ = 1∣*J*_*n*,*i*_ = 1) and *P*(*M*_*n*,*j*_ = 1) terms separately to create a single composite Ecosystem matrix (using data from 1984-2010).

Building the *P*(*M*_*n*,*j*_ = 1∣*J*_*n*,*i*_ = 1) term:

1. For each country and product pair, we calculate RCA values (top row in Fig. 8).

**Fig. 8:**
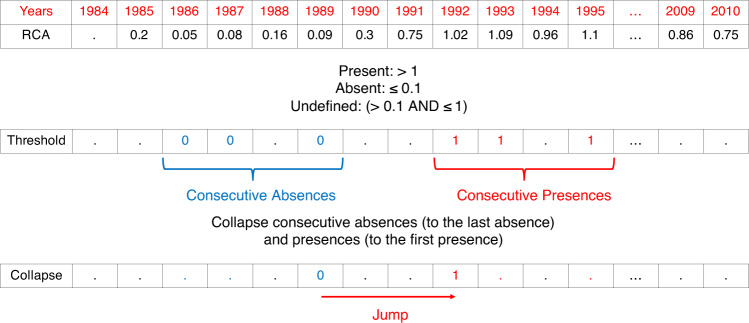
Example of a jump. We calculate RCA values for each year (top row). We convert the RCA values to absences and presences based on corresponding thresholds (middle row). We merge consecutive absences to the latest year and consecutive presences to earliest year. A jump is defined as transforming an absence to presence.
2. We designate a product absent if its RCA value is below *τ*_0_ (= 0.1 in Fig. 8) and present if its RCA value is above *τ*_1_ (=1 in Fig. 8). If the RCA value is between these two thresholds we designate this product undefined. If the country-product pair is missing for that year, we also designate it undefined (middle row in Fig. 8).
3. We collapse all consecutive absences—and absences interspaced with undefined values—to the latest absence (bottom row in Fig. 8).
4. We collapse all consecutive presences—and presences interspaced with undefined values—to the earliest presence (bottom row in Fig. 8).
5. After collapsing, we are guaranteed to have a single absence followed by at most a single presence. After the presence, however, another absence could be present. A jump occurs when a country transitions from an absence to a subsequent presence (bottom row in Fig. 8).
6. For a product *i*: we search for the set of countries ${{\mathcal{K}}}_{i}$ in which it appeared. For each of these countries, we detect which other products *j* were present in the jump start year. A product *j* was present in the start year if its RCA value was greater than *τ*_1_.
7. For each *i* and *j*, we compute the total number of presences of each product *j* (given an appearance of product *i*), and divide it by the number of appearance countries (e.g. the size of set ${{\mathcal{K}}}_{i}$).

Building the *P*(*M*_*n*,*j*_ = 1) term:

1. For each product *j*, we compute the total number of presences of each product *j* across all countries (i.e., *RCA*_*n*,*j*_ > *τ*_1_) and years.
2. We divide the total number of presences of product *j* across all years by the total number of countries (each country is counted once for each year it appears in the sample).

Finally, the Ecosystem is a log of the ratio of the *P*(*M*_*n*,*j*_ = 1∣*J*_*n*,*i*_ = 1) and *P*(*M*_*n*,*j*_ = 1) terms.

Notes:

- Unless otherwise specified, we set standard values for parameters for absence and presence: *τ*_0_ = 0.1 and *τ*_1_ = 1.
- Following Hausmann et al.^2^, we restrict our sample to countries with population greater than 1.2 million and total exports of at least $1 billion in 2008. There are also countries with known data reporting issues that were removed by Hausmann et al.^2^. The sample reduces to 125 countries for the Ecosystem matrix computation.
- The full SITC Rev.2 has 786 4-digit products in 1984. We omit 6 products with one-digit code 9 (‘Commodities and transactions not classified elsewhere in the SITC’), and drop to 780 products. Then we drop products that do not constitute more than one in one millionth of world trade and have at least 5 million USD exports in all 33 years, which reduces the number of products to 756. Eliminated products are very small in terms of export volume, and create spurious jumps.
- The definition of RCA enables small countries to surpass the presence threshold easily. To minimize noise, we converted presences (*RCA* > 1) to undefined if the countries’ export in the product is less than $10 thousand or the country exports less than one in ten thousandth of the product. Overall, in 33 years, we have 474,494 presences and this change affects 4801 of them (~1%).

### Predicting product appearances

A country *n* has capabilities *c*_*n*_, and products $j\in {{\mathcal{J}}}_{n}$. We want to compute the probability that country *n* will acquire the missing capabilities for the appearance of product *i*:

$$P({J}_{n,i}=1)={q}^{\parallel {{\bf{p}}}_{{\bf{i}}}\parallel -{{\bf{c}}}_{{\bf{n}}}\cdot {{\bf{p}}}_{{\bf{i}}}}$$

We do not know which capabilities country *n* already has, but we can proxy for them by looking at the capabilities of products already present in the country:

$${{\bf{c}}}_{{\bf{n}}}={\mathbb{1}}\left(\sum _{j\in {{\mathcal{J}}}_{n}}{{\bf{p}}}_{{\bf{j}}}\right)$$

where the function ${\mathbb{1}}$ sets the entry of a vector to be 1 if the corresponding entry of the input vector is greater than or equal to 1, i.e., the entry *k* of **c**_**n**_ is 1 if at least one product present in country *n* requires capability *k*.

**Fig. 9:**
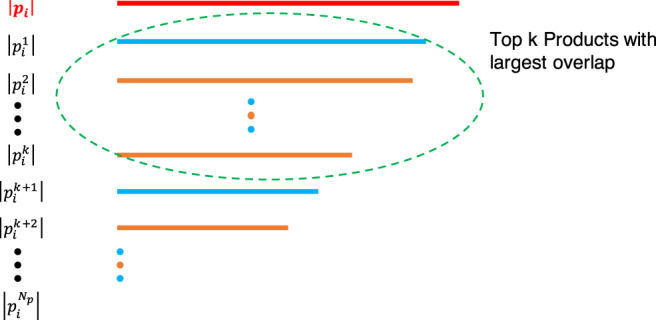
Capability overlaps and building density measures. The length of the capability vector for product *i*, ∥**p**_**i**_∥, is unknown. But we have estimates of the overlaps, the $\parallel {{\bf{p}}}_{{\bf{i}}}^{{\bf{j}}}\parallel$’s. The blue products are not made by the country whereas the orange products are.

For each product *i*, we do not know the length of its capability vector, ∥**p**_**i**_∥, but using the Ecosystem entries, we obtain estimates for the overlaps, $\parallel {{\bf{p}}}_{{\bf{i}}}^{{\bf{j}}}\parallel$’s. We will assume that these overlaps between the products are uniformly distributed. Under this assumption, we can estimate ∥**p**_**i**_∥ as the maximum of the $\parallel {{\bf{p}}}_{{\bf{i}}}^{{\bf{j}}}\parallel$’s. Then the maximum likelihood estimator of the maximum statistic for a uniform distribution is:

$$\parallel \widehat{{{\bf{p}}}_{{\bf{i}}}}\parallel =\frac{{N}_{p}+1}{{N}_{p}}\mathop{\max }\limits_{j}\parallel {{\bf{p}}}_{{\bf{i}}}^{{\bf{j}}}\parallel$$

where *N*_*p*_ is the number of products. Figure 9 depicts this process. Hence, we estimate the number of capabilities needed for *i* by computing its maximum overlap with all other products *j*.

Country *n* makes only a subset of these products and from this subset we can estimate the **p**_**i**_.**c**_**n**_ term. The maximum likelihood estimator (up to the same multiplicative factor as above) for the overlap **p**_**i**_.**c**_**n**_ is then:

$$\widehat{{{\bf{p}}}_{{\bf{i}}}{\boldsymbol{.}}{{\bf{c}}}_{{\bf{n}}}}=\mathop{\max }\limits_{j\in {{\mathcal{J}}}_{n}}\parallel {{\bf{p}}}_{{\bf{i}}}^{{\bf{j}}}\parallel$$

This is the maximum overlap between product *i* and any product *j* which is present in country *n*.

Empirically, we estimate $\parallel \widehat{{{\bf{p}}}_{{\bf{i}}}}\parallel$ as $\mathop{\max }\nolimits_{j}{\hat{E}}_{i,j}$ and $\widehat{{{\bf{p}}}_{{\bf{i}}}{\boldsymbol{.}}{{\bf{c}}}_{{\bf{n}}}}$ as $\mathop{\max }\nolimits_{j\in {{\mathcal{J}}}_{n}}{\hat{E}}_{i,j}$. Therefore, we estimate the likelihood of an appearance of a product *i* in country *c* as

$${d}_{n,i}^{E}={q}^{\mathop{\max }\nolimits_{j}{\hat{E}}_{i,j}-\mathop{\max }\nolimits_{j\in {{\mathcal{J}}}_{c}}{\hat{E}}_{i,j}}$$

where *q* is the probability of acquiring a new capability, and ${{\mathcal{J}}}_{n}$ is the set of products present in country *n*. In order to reduce noise, we take the mean value over the top *k* = 25 entries for each $\mathop{\max }\nolimits_{j}{\hat{E}}_{i,j}$ and $\mathop{\max }\nolimits_{j\in {{\mathcal{J}}}_{n}}{\hat{E}}_{i,j}$. The robustness of our results in terms of parameter *k* is given in Supplementary Note 3.4 of the Supplementary Information.

### The product space

The Product Space^3^ is a network that was proposed to model the process of industrial diversification of nations. Similar to the Eco Space, nodes represent products, and edges are intended to capture capability overlap. The Product Space is built from a cross-section of data—as opposed to the time-series data required to build the Eco Space. The edge weight between two nodes is estimated using a measure of co-export—i.e., a pair of products is connected by an edge if they are exported by a similar set of countries. It has been shown that the Product Space is a good predictor of product appearances^3,31^.

Hidalgo et al.^3^ define the Product Space as a matrix *P* such that

$${P}_{i,j}=\frac{\sum _{n}{M}_{n,i}{M}_{n,j}}{\max (\sum _{n}{M}_{n,i},\sum _{n}{M}_{n,j})}$$

where *M*_*n*,*i*_ = 1 if country *n* makes product *i*, and 0, otherwise. The logic behind this approximation is that if a pair of products is co-exported by a large subset of countries, then these products must require a similar capability base.

Consequently, countries are expected to move into industries which are close or similar to activities they are already successful at. From a network perspective, this is equivalent to saying that the probability of a product appearance in the future is dependent on the RCA that the country currently enjoys in neighbouring products. Mathematically, we write the Product Space density of product *i* in country *n* as

9 $${d}_{n,i}^{P}=\frac{{\sum }_{j}{P}_{j,i}{M}_{n,j}}{{\sum }_{j}{P}_{j,i}}.$$

where the matrix *P* represents the network proximity or adjacency matrix for the Product Space as defined above.

### Probit model

We perform a standard Probit regression for the probability of a product appearance of the form:

10 $${J}_{n,i}={\mathbf{\Phi }}(\alpha +{\beta }_{E}{d}_{n,i}^{E}+{\beta }_{P}{d}_{n,i}^{P}+{\gamma }_{i}+{\eta }_{n})$$

where the binary variable *J*_*n*,*i*_ is defined by Eq. (7), **Φ** is a normal cumulative distribution function, *d*^*E*^ corresponds to the Ecosystem density, and *d*^*P*^ corresponds to the Product Space density, and *γ*_*i*_ and *η*_*n*_ are product and country fixed effects respectively.

We construct the Ecosystem for years 1984–2009, and use RCA values from the year 2010, to compute the density metrics for both the Eco Space and the Product Space. Our dependent variable is defined for appearances during the 6-year period 2010–2016. Note that we condition on the product being absent at the start of the period, e.g., we only include country-product pairs that were absent in 2010.

In order to quantify the predictive power of each density metric, and their combination, we compute the AUC or Area Under the Curve of the ROC (Receiver Operating Characteristic). The ROC curve plots the rate of true positives of a continuous prediction criterion as a function of the rate of false positives. The area under the curve (AUC) statistic is equivalent to the Mann–Whitney statistic (the probability of ranking a true positive ahead of a false positive in a prediction criterion). By definition, a random prediction will find true positives and false positives at the same rate, and hence will result in an AUC = 0.5, whereas AUC = 1 for a perfect prediction.

## Supplementary information

Supplementary Information

## Data Availability

All data that we use in this study is publicly available. The trade data is available from the Atlas of Economic Complexity Dataverse, http://dataverse.harvard.edu/dataverse/atlas. Country level indicators were obtained from the World Development Indicators database, http://datatopics.worldbank.org/world-development-indicators/. The shape files for the world maps were downloaded from https://thematicmapping.org/downloads/world_borders.php. The shape files were not altered, and only used for mapping levels of several variables. The shape files are licensed under Creative Commons Attribution-Share Alike License (https://creativecommons.org/licenses/by-sa/3.0/).

## Code availability

The analysis in this study was done using Stata and Matlab. The Stata and Matlab code is available upon request. The network layout was generated using Gephi (https://gephi.org/). The communities in the network were identified using the Partition Stability algorithm (http://wwwf.imperial.ac.uk/~mpbara/Partition_Stability/).
