# Supplementary material for: Productive Ecosystems and the arrow of development
Source: Nat Commun. 2021 Mar 5;12:1479. doi: 10.1038/s41467-021-21689-0 (PMC7977064; doi:10.1038/s41467-021-21689-0)
Supplement: Supplementary file 1 — Supplementary Information [file 41467_2021_21689_MOESM1_ESM.pdf]

–Supplementary Information–
Productive Ecosystems and the Arrow of Development

Neave O'Clery, Muhammed A. Yıldırım & Ricardo Hausmann

Supplementary Note 1 Sensitivity to Thresholds and Time

An appearance of product i in country n is defined as:

$$J_{n,i} = \begin{cases} 1 & \text{if } R_{n,i} \leq \tau_0 \text{ in } t_0 \text{ and } R_{n,i} > \tau_1 \text{ in } t_1 \\ 0 & \text{otherwise} \end{cases}$$

In our main specification, we use thresholds of $\tau_0 = 0.1$ and $\tau_1 = 1$. How sensitive are our results to these thresholds?

In Supplementary Figure 1, we increase the threshold τ_0 from 0 to 0.30 in increments of 0.05 and τ_1 from 0.7 to 1.30 in increments of 0.1. We then recalculate the Ecosystem matrices for 49 different combinations.

Supplementary Figure 1a shows the correlation between the vectorized versions of all Ecosystem matrices. The axes are sorted first according to τ_1 and then τ_0. As is evident in the figure, the correlations are higher for similar values of τ_0 and τ_1. Correlations around the diagonal are close to 1 and the correlations decrease as we move away from the diagonal. A key exception is $\tau_0 = 0$. An absence threshold of 0 means that a country exporting any non-zero amount will not appear as absent, and, hence, countries misreporting or re-exporting goods will not be eligible for a jump. Hence the correlation between $\tau_0 = 0$ and any other combination of thresholds is low.

Supplementary Figure 1b shows the correlations between the Ecosystem input vectors, and

Supplementary Figure 1c shows the correlations between the Ecosystem size vectors. In both correlation matrices, we observe very high correlation values close to 1. The correlations are somewhat higher for the Ecosystem input vectors. Again, $\tau_0 = 0$ is an exception, but even in this case correlations exceed than 70%.

Checking the sensitivity of our Ecosystem matrix with regards to time is not trivial as we require data covering a significant period of time in order to build our matrix. The simplest approach would be to split the full period into two sub-periods, namely 1984-1999 and 2000-2016. But the problem with this approach is that the nature of the global export basket differs between these two time periods. For instance computers, mobile phones, etc. become important goods in the second period. Hence, the dynamics of new product transitions is expected to be very different in the two sub-periods. In order to circumvent these issues and to test the stability of the Ecosystem matrix over time, we split the sample into odd and even years. The correlation between the full Ecosystem matrix is 60%, whereas the correlation between the Ecosystem input and Ecosystem size vectors is 97% and 90%, respectively.

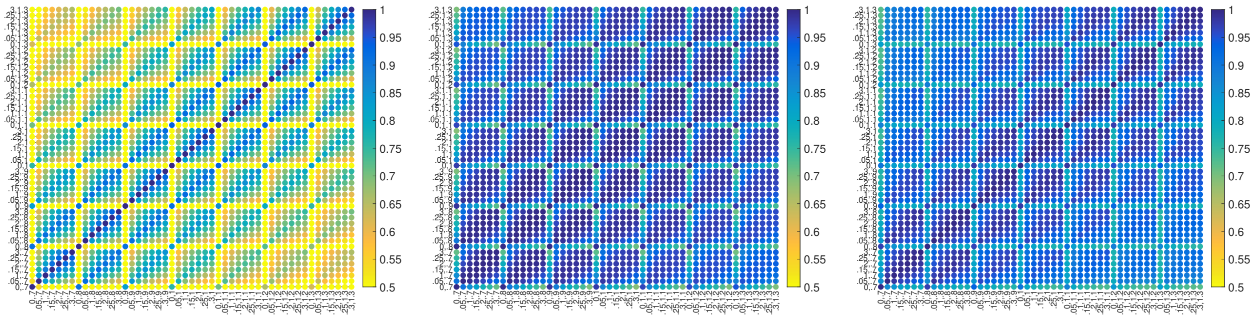

(a) All Ecosytem

(b) Ecosystem Input

(c) Ecosystem Size

Supplementary Figure 1: Correlations of Ecosystem matrix for various absence and presence thresholds.Each entry in the above matrices correspond to a correlation between vectors of Ecosystem entries for various absence and presence thresholds. The first number in the row and column labels corresponds to the absence threshold (τ_0) which increases from 0 to 0.3 in increments of 0.05. The second number corresponds to the presence threshold (τ_1) which increases from 0.7 to 1.3 in increments of 0.1. This results in 49 different combinations of absence and presence thresholds. The labels are first sorted according to τ_1 and then by τ_0.

As a second exercise to test the temporal stability of the Ecosystem matrix, we start with the Ecosystem matrix from 1984-1999 and sequentially add additional years until we reach 2016, the final year of our dataset. Since we allow multiple jumps by a country to a product, additional years have the potential to substantially alter the Ecosystem matrix. Supplementary Figure 2a

shows that there is a higher correlation between close years and a relatively lower correlation as we move further away from the diagonal. The correlations are significantly higher with values close to 1 if we calculate the correlation between the Ecosystem input and Ecosystem size vectors (Supplementary Figures 2b and 2c).

As a benchmark, we can also check similar correlations for the Product Space. Supplementary Figure 3a shows the correlation between different presence threshold values (τ_1 from 0.7 to 1.3) for the Product Space (2016 data). The correlation values range from 0.27 to 0.95. When we compare Product Spaces between different years using $\tau_1 = 1$ (Supplementary Figure 3b), we observe values ranging from 0.37 to 0.85 with higher values closer to the diagonal.

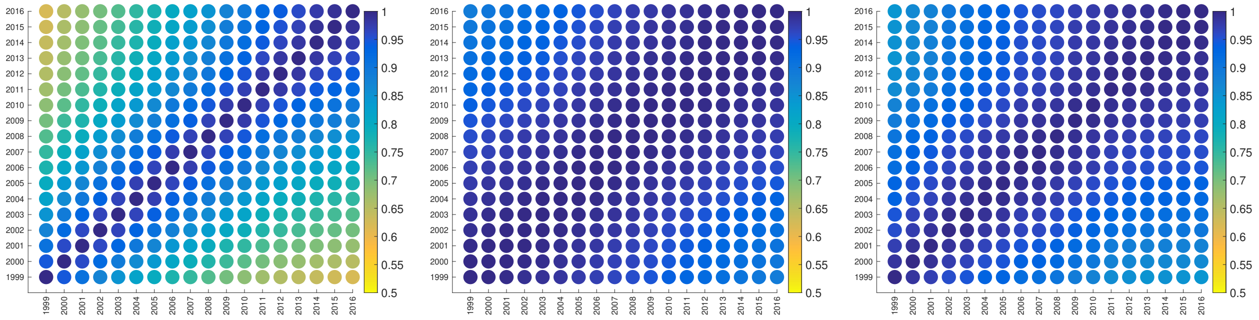

(a) All Network (b) Ecosystem Input (c) Ecosystem Size

Supplementary Figure 2: Stability of Ecosystem Matrix over Time.Each entry corresponds to the correlation between vectors of Ecosystem entries for Ecosystems starting in 1984 and ending in the specified year as indicated by the row and column labels. The absence and presence thresholds used here are $\tau_0 = 0.1$ and $\tau_1 = 1$, respectively.

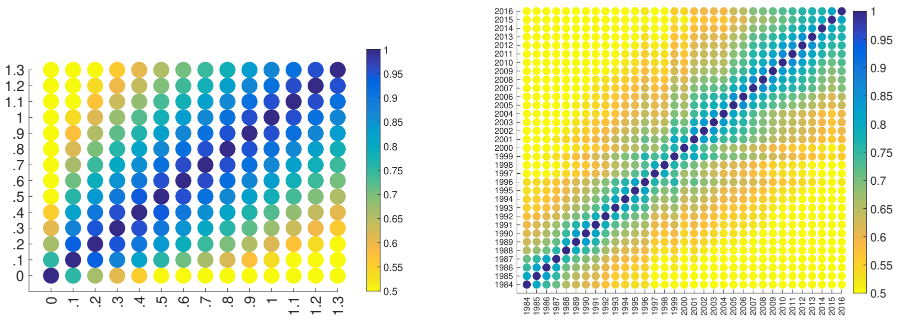

(a) For different presence thresholds (b) Between years

Supplementary Figure 3: Correlations of Product Space Matrix. On the left hand side, (a), we show the correlations between Product Spaces for different presence thresholds (τ_1), ranging from 0.7 to 1.3 in increments of 0.1. Rows and columns are labeled with the corresponding presence threshold values. On the right hand side, (b), we show the correlations between Product Spaces obtained for different years, indicated by the row and column labels with a presence threshold of $\tau_1 = 1$.

Supplementary Note 2 Jumps by Countries and Products

We calculate the Ecosystem matrix by observing jumps by many countries and products. Which countries jump more, and are there any specific characteristics of the products that we observe many jumps for?

Supplementary Figure 4 shows the number of jumps for products and countries. In Supplementary Figure 4a we observe that most jumps occur for the products with medium export volumes. In Supplementary Figure 4b, we see that most jumps happen in countries with relatively small export size like Jordan and Lebanon. The importance of the export size is expected as the formula for RCA can be written as:

$$R_{n,i} = \frac{X_{n,i} / \sum_k X_{k,i}}{\sum_j X_{n,j} / \sum_k \sum_j X_{k,j}}$$

The numerator, $X_{n,i} / \sum_k X_{k,i}$, is the share of country n in industry i, whereas the denominator is the share of country n in total world trade. If country n is small, its share of world trade will also be small. Hence $R_{n,i} > 1$ even for relatively small values of $X_{n,i}$. If we increase the presence threshold, τ_1, however it will become more difficult for larger countries to exceed the threshold. Finally, export volume alone is not the sole determinant of a large number of jumps. This requires dynamic exporting behaviour.

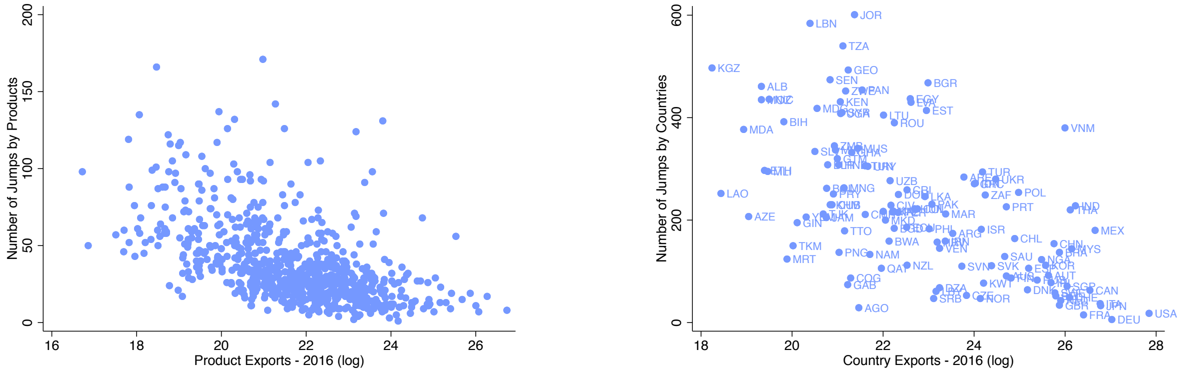

(a) Products

(b) Countries

Supplementary Figure 4: Number of jumps. Panel (a) shows number of jumps for each product as a function of total product export. Panel (b) shows the number of jumps made by countries as a function of their export size.

In Supplementary Figure 4, we allow multiple jumps to a single product. One way to decrease the number of jumps, and limit the number if jumps from smaller countries, would be to allow a single jump per country. To do that, we only consider the first jump made by a country to a product. Supplementary Figures 5a and 5d show that, as expected, this change decreases the overall number of jumps. It does not however significantly alter the rankings of the countries and products in terms of the number of jumps.

Another way to limit the effect of countries with smaller export size is to apply a more stringent definition of a jump. In our main specification, we assume a country jumps to a product with a single year below $R_{n,i} \leq \tau_0$ and another year above $R_{n,i} > \tau_1$. We can make this criterion more stringent by forcing countries to be below $R_{n,i} \leq \tau_0$ for at least two consecutive years and above $R_{n,i} > \tau_1$ for at least two consecutive years. In Supplementary Figures 5b and 5e we allow multiple jumps with this specification and show that having two-years of absence followed by two-years of presence does not significantly change the ranking of countries and products. Supplementary Figures 5c and 5f compares the number of jumps for our original single jump set-up and our two-year jump set-up.

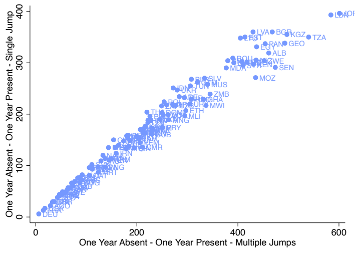

(a) Countries: One year absence & presence, single-jump.

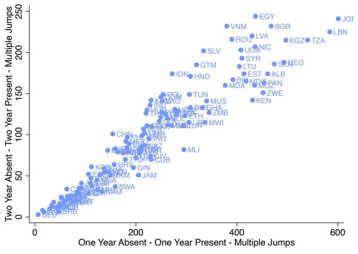

(b) Countries: Two year absence & presence, multi-jumps

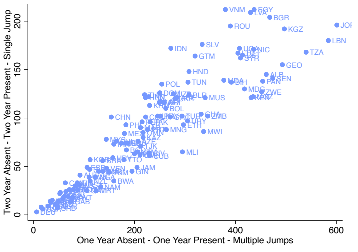

(c) Countries: Two year absence & presence, single-jump

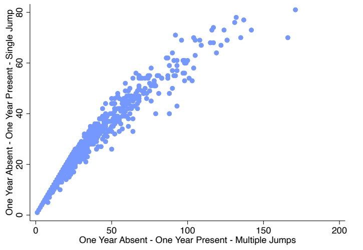

(d) Products: One year absence & presence, single-jump.

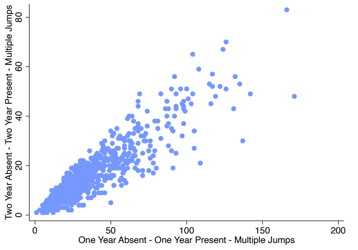

(e) Products: Two year absence & presence, multi-jumps

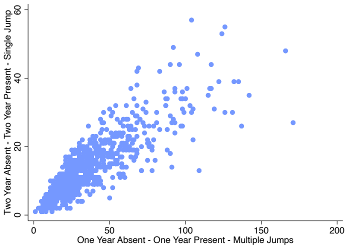

(f) Products: Two year absence & presence, single-jump

Supplementary Figure 5: Comparison of Jumps for Countries and Products. Top row shows the jump by countries and bottom row shows the jumps by products as a function of export volumes.

Supplementary Note 3 Robustness Checks for the Regression Results

First, a step-by-step explanation of how we build the Ecosystem density is provided. Suppose we want to estimate the likelihood that country n will jump to product i, which country n is not currently manufacturing.

1. The country capability endowment vector $\mathbf{c_n}$ and product capability requirement vector p_i are unknown. The likelihood of the jump is determined by the difference $\|\mathbf{p_i}\| - \mathbf{c_n}^T \cdot \mathbf{p_i}$, where ^{T} denotes the transpose. We will drop the transpose sign for decluttering the notation.

2. From the predicted Ecosystem matrix, $\mathbf{\hat{E}}$, we have an estimate for the overlap between any product with product i, and $\mathbf{\hat{E}_{i,\cdot}} \equiv (\|\mathbf{p_i^1}\|, \|\mathbf{p_i^2}\|, \ldots, \|\mathbf{p_i^N}\|)$ represents this overlap. For instance, $\|\mathbf{p_i^1}\|$ estimates the capability overlap between product 1 and product i. Each of these values is smaller than $\|\mathbf{p_i}\|$, i.e., $\forall j \in 1, \ldots, N \quad \|\mathbf{p_i^j}\| \leq \|\mathbf{p_i}\|$.

3. We wish to estimate $\|\mathbf{p_i}\|$ using our set of overlaps. If we assume a uniform distribution, then the maximum of $(\|\mathbf{p_i^1}\|, \|\mathbf{p_i^2}\|, \ldots, \|\mathbf{p_i^N}\|)$ would be the maximum likelihood estimate up to a constant.

4. For $\mathbf{c_n} \cdot \mathbf{p_i}$ term, we consider only the overlap between product i and products present in country n. Similar to the argument above, the maximum of the set $\{\|\mathbf{p_i^j}\|\}$ for all products j produced in country n is our estimate of $\mathbf{c_n} \cdot \mathbf{p_i}$ up to a constant.

5. In order to smooth our estimates, we use the average of the top k values, rather than selecting a single maximum value in the previous two points. Selecting 'nearest neighbours' has been a common practice especially in the machine learning literature. Dude *et al.*[1] gives $\sqrt{N}$ as a rule of thumb in their book titled 'Pattern classification'. In our case, we have 756 products and $\sqrt{756} \approx 27$, which is close to the number we currently use of $k = 25$. In Section C.3, we analyze the robustness of our results to this parameter choice.

Supplementary Note 3.1 Distribution of Ecosystem Density and Product Space Density

The Ecosystem density measure is the key independent variable that we test in our regressions in Table 2. Does this measure vary systematically across countries?

In Supplementary Figure 6a we show the box plot of the normalized Ecosystem density for all the countries that are present in our dataset for the year 2010. As a comparator, in Figure 6b we show the box plot of the Product Space density proposed by Hidalgo *et al.*[2]. In both figures, the countries are sorted according to their ECI values. The Ecosystem density does not exhibit large variation across countries relative to the variation in the Product Space density, which appears to depend on the ECI of the countries.

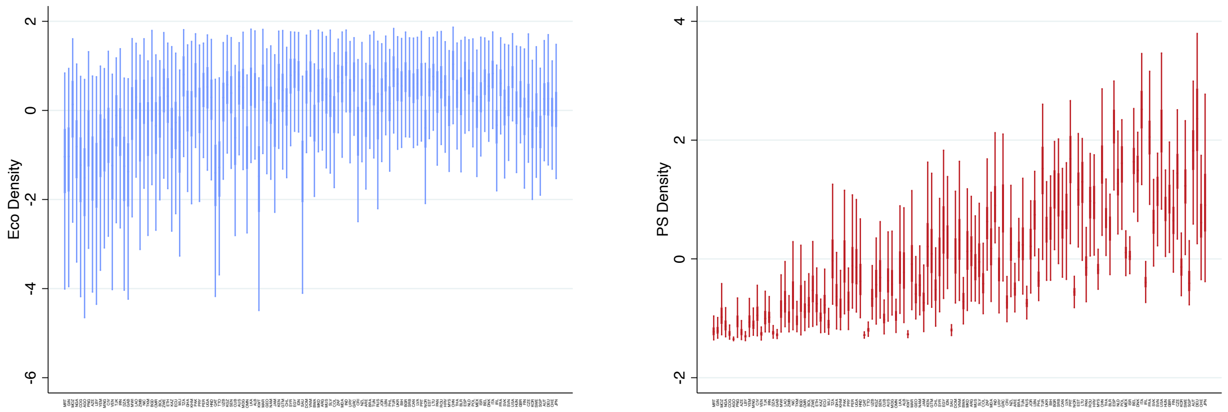

(a) Ecosystem Density (Normalized)

(b) Product Space Density (Normalized)

Supplementary Figure 6: Distribution of Ecosystem Density and Product Space Density over countries.In both figures, we normalize the density values by subtracting the mean and dividing by the standard deviation. The countries on the x-axes are sorted according to their ECI values. The thicker portion of each line corresponds to the 25th and 75th percentile of the density distributions for each country. The upper and lower adjacent values are calculated using Stata's default box plot option. For clarity we are not showing outliers.

Supplementary Note 3.2 Area Under the Curve (AUC) Measure

In Table 2, we assess whether the Ecosystem Density measure is predictive of jumps. At time $t_0 = 2010$, all countries that do not make a product or have RCA less than $t_0 = 0.1$ in a product are in our sample set. Some of the products will appear in the future but some will not.

In order to investigate the discriminatory power of the Ecosystem Density, we build a Receiver

Operating Characteristic (ROC) curve. ROC curves have the TPR (true positive rate = true positives/positives) on the Y axis and the FPR (false positive rate = false positives/negatives) on the X axis. A ROC curve highlights the relative tradeoffs between costs (false positives) and benefits (true positives) of a classifier. Suppose we set a threshold for the Ecosystem Density and predict that any country-product pair above that value will be positive and below that will be negative. From this we can calculate the TPR and FPR. By moving the threshold from the minimum value to maximum value of the Ecosystem Density, we can build a ROC curve. An important feature of a ROC curve is that the area under it is equivalent to the probability that the classifier ranks a randomly chosen positive instance higher than a randomly chosen negative instance.[3] The Area Under the Curve (AUC) measure is best used for comparing multiple methods for their discrimination power.

Figure 7 shows the ROC curves for the Ecosystem Density and the Product Space Density (corresponding to columns 1 and 4 of Table 2). As is evident from the figure, the Ecosystem Density exhibits a higher predictive power throughout the range. The dashed line corresponds to a random classifier such as a coin toss.

What does an AUC value of 0.715 tell us about the performance of our classifier? On page 177 of the highly influential book entitled "Applied Logistic Regression" by Hosmer Jr *et al.*[4] the authors list the following rule of thumb for AUC values:

$$\text{If} \begin{cases} \text{AUC} = 0.5 & \text{No discrimination. Same as coin flip} \\ 0.5 < \text{AUC} < 0.7 & \text{Poor discrimination.} \\ 0.7 \leq \text{AUC} < 0.8 & \text{Acceptable discrimination} \\ 0.8 \leq \text{AUC} < 0.9 & \text{Excellent discrimination} \\ \text{AUC} \geq 0.9 & \text{Outstanding discrimination} \end{cases}$$

Hence, an AUC value of 0.715 for the Ecosystem Density is acceptable according to these rules. Whereas, the AUC value for the Product Space is considered to exhibit poor discrimination. When we add country and product fixed effects to control for unobserved characteristics, we obtain AUC

levels higher than 0.8 (Column 4 of Table 2).

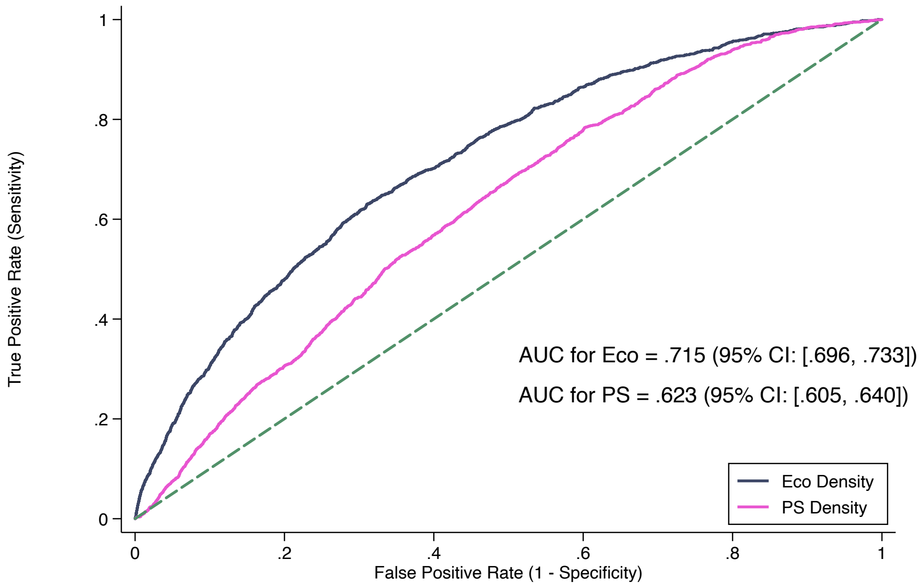

Supplementary Figure 7: Receiver Operating Characteristic Curve for demonstrating the predictive power of the ecosystem density and product space density.

Is an AUC value of 0.715 significant? According to Hanley and McNeil[5], the distribution of AUC values follows an approximately normal distribution for large samples under some normality assumptions. The authors report the standard error of AUC measure to be:

$$SE(\text{AUC}) = \sqrt{\frac{\text{AUC}(1-\text{AUC}) + (P-1)\left(\frac{\text{AUC}}{2-\text{AUC}} - \text{AUC}^2\right) + (N-1)\left(\frac{2\text{AUC}^2}{1+\text{AUC}} - \text{AUC}^2\right)}{PN}}$$

where P denotes the number of positives and N denotes the number of negatives. The 95% confidence interval for the AUC based on this standard error is $[0.696, 0.733]$ for the Ecosystem Density and $[0.605, 0.640]$ for the Product Space Density. Hence, we can reject the null hypothesis that the AUC values are equal for the Ecosystem Density and the Product Space Density at the 95% confidence level.

Supplementary Note 3.3 Sensitivity of explanatory power to Ecosystem construction period

Absence and presence thresholds related to jumps are also important for the regression analysis. In Supplementary Figure 8, we compare the AUC values corresponding to various threshold combinations for the Ecosystem and Product Space densities. Overall, the Ecosystem density outperforms the Product Space density for any choice of threshold.

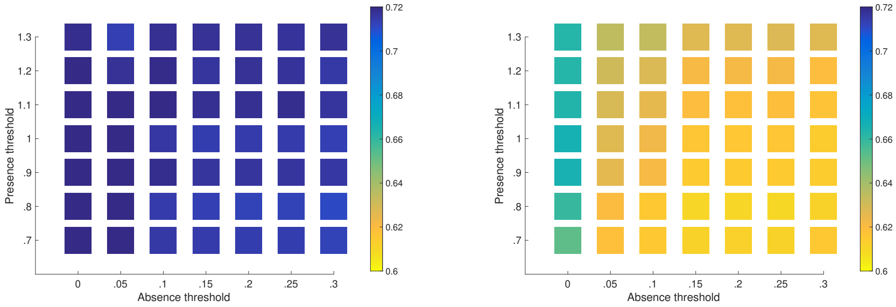

(a) Ecosystem (b) Product Space

Supplementary Figure 8: AUC results for various absence/presence thresholds.Each entry in the above matrices corresponds to the AUC value for the probit regressions with various combinations of absence and presence thresholds. The columns correspond to the absence threshold (τ_0), ranging between 0.0 to 0.3, in increments of 0.05. The rows correspond to the presence threshold (τ_1), ranging from 0.7 to 1.3, in increments of 0.1. In total, we have 49 combinations of absence and presence thresholds. The corresponding Ecosystems were calculated using the same (τ_0, τ_1) pair and the Product Space is calculated using the corresponding τ_1.

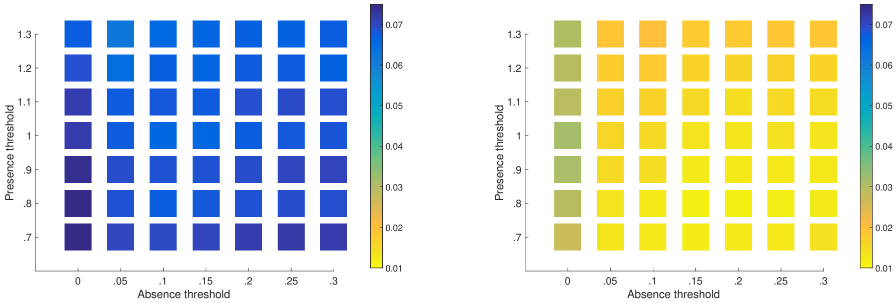

(a) Ecosystem (b) Product Space

Supplementary Figure 9: R^2 values for various absence/presence thresholds.Each entry in the above matrices corresponds to the pseudo-R^2 value for the probit regressions with various combinations of absence and presence thresholds. Please refer to the note under Supplementary Figure 8 for explanation of each cell.

In Supplementary Figure 9, we repeat the same exercise, but instead of AUC metric, we use the pseudo-R^2 values to compare the predictive power of Ecosystem density and the Product Space density. Pseudo-R^2 captures the contribution of the variable of interest to the explanatory power

in the probit regressions. As expected, the overall patterns are quite similar with Supplementary Figure 8. Here, we observe 4 to 5 times increase in explanatory power with Ecosystem density (except when the absence threshold is 0, for which we see close to 2.5 times increase).

Supplementary Note 3.4 Sensitivity of Results to the Neighbourhood Size

In our main specification, we choose the top 25 product overlaps to estimate the capability gap in our density measure. This is in order to minimize the effects of errors in the export data: if we choose very few products, noise might affect our results. On the other hand, if we choose many products, then our estimate of the capability overlap would include products with relatively low overlap with our product of interest.

In Supplementary Figure 10, we re-run our main specification while varying parameter k, the number of products selected in our overlap estimates. In line with our expectations, including a very low or high number of neighbours decreases our predictive power. Within the range of 20 to 60 products, we have relatively stable predictive power.

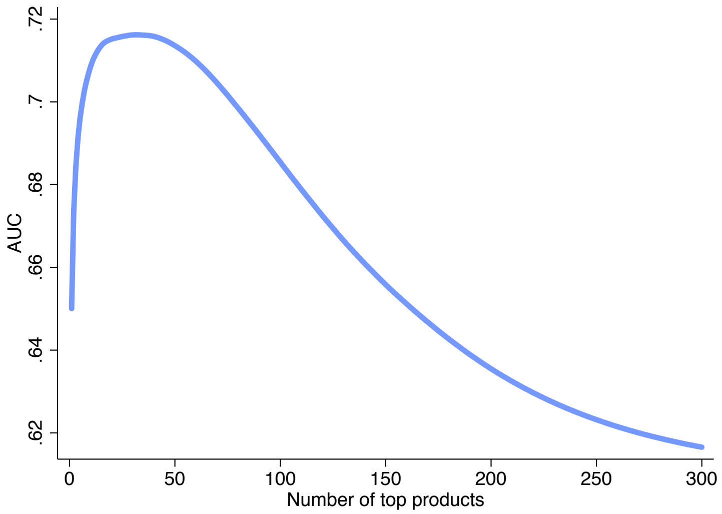

Supplementary Figure 10: AUC results obtained from jump predictions for various neighbourhood sizes while calculating the ecosystem density.

Supplementary Note 3.5 Sensitivity to Country and Product Groups

In order to further investigate the sensitivity of our regression results, here we re-run our prediction analysis splitting our sample by a range of country and product characteristics.

First, in Supplementary Table 1 we show the results for various subsets of countries. In Columns 1 and 2, we split countries by income level into high-income (per capita GDP above or equal to median per capita GDP) and low-income (per capita GDP below median per capita GDP). The difference in the coefficient of Ecosystem density between the high- and low-income samples is not statistically different. As expected, there are more opportunities to jump for low-income countries (higher number of observations) and there are more realized jumps. However the AUC is higher for high-income countries, implying that these countries take better advantage of the adjacent opportunities as captured by their densities.

VARIABLES	(1)<br>High Inc.	(2)<br>Low Inc.	(3)<br>High ECI	(4)<br>Low ECI	(5)<br>High Exp.	(6)<br>Low Exp.
Eco Density	8.9591***	9.6140***	14.1155***	9.1016***	7.2810***	10.3293***
	(0.6666)	(0.4872)	(0.9391)	(0.4226)	(0.6467)	(0.4929)
Constant	-10.0820***	-10.5711***	-14.9389***	-10.0922***	-8.6327***	-11.1924***
	(0.6162)	(0.4527)	(0.8833)	(0.3897)	(0.5963)	(0.4582)
Observations	19,324	30,028	13,360	35,992	19,578	29,774
Number of App.	560	1271	528	1303	451	1380
Country FE	No	No	No	No	No	No
Product FE	No	No	No	No	No	No
Pseudo R2	.065	.062	.071	.069	.049	.067
AUC	.719	.709	.719	.720	.696	.714

Robust standard errors in parentheses
*** $p<0.01$, ** $p<0.05$, * $p<0.1$

Supplementary Table 1: Sensitivity of Results for Different Country Groups. Countries are divided into two groups based on their income, ECI and export volumes.

In Columns 3 and 4, we divide countries by complexity level into high-complexity (ECI above or equal to 0) and low-complexity (ECI below 0). Low-complexity countries are more likely to jump into new products, but the Ecosystem density coefficient is higher for high-complexity countries. AUC levels are quite similar for both the high- and low-complexity samples, indicating that ECI is capturing more about the sophistication of a country's capability base than its income. ECI and per capita GDP are highly correlated, but there are significant outliers, mostly natural resource

rich countries. Hence, the difference between the first two columns and the next two columns can be attributed to these countries.

In Columns 5 and 6, countries are split based on export levels, into high-export (export levels above or equal to median) and low-export (export levels below median). As discussed previously, as the export size of countries increases, it becomes more difficult to satisfy the RCA-based criteria for a jump. As a result, the AUC, as well as the coefficient of the Ecosystem density variable, is lower for high export countries.

We then conduct a similar analysis for different product categories, as shown in Supplementary Table 2. First, we want to address the issue that the predictive power of our results might stem from the classification system. To give an example, let's take two products, namely men's shirts and women's shirts, which are classified separately. If our methodology relies on information from men's shirts to predict a jump to women's shirts, then in another classification system it would not work. In other words, this would imply that our methodology captures some artifacts of the classification system. Although our strict enforcement of precedence between products while defining the Ecosystem matrix might dampen this issue, we wish to formally test this effect.

VARIABLES	(1)<br>No 3-Dig.	(2)<br>No 2-Dig.	(3)<br>Manuf.	(4)<br>Non Manuf.	(5)<br>High PCI	(6)<br>Low PCI	(7)<br>High Ubq.	(8)<br>Low Ubq.	(9)<br>High Vol.	(10)<br>Low Vol.
Eco Density	9.4131***	9.3023***	9.2981***	9.6221***	7.8051***	11.3535***	11.1227***	8.3856***	7.9598***	9.9944***
	(0.3920)	(0.3894)	(0.4877)	(0.6796)	(0.4975)	(0.5853)	(0.6017)	(0.4948)	(0.5851)	(0.5318)
Constant	-10.4273***	-10.3276***	-10.3375***	-10.5916***	-8.9947***	-12.1898***	-11.9884***	-9.4996***	-9.1644***	-10.9232***
	(0.3637)	(0.3614)	(0.4508)	(0.6337)	(0.4577)	(0.5464)	(0.5606)	(0.4570)	(0.5365)	(0.4964)
Observations	49,352	49,352	32,350	17,002	27,093	22,259	20,711	28,641	22,885	26,467
Number of App.	1831	1831	1045	786	773	1058	902	929	587	1244
Country FE	No	No	No	No	No	No	No	No	No	No
Product FE	No	No	No	No	No	No	No	No	No	No
Pseudo R2	.064	.063	.072	.048	.059	.062	.066	.062	.055	.059
AUC	.714	.712	.727	.685	.710	.703	.710	.715	.703	.702

Robust standard errors in parentheses
*** p<0.01, ** p<0.05, * p<0.1

Supplementary Table 2: Sensitivity of Results for Different Product Groups. In first two columns, we do not use information from products that have same two and three leftmost digits while calculating ecosystem density. In the next columns we divide products based on manufacturing, PCI, ubiquity and export volumes.

In order to do this, we construct a version of the Ecosystem in which we do not allow any product with the same three-digit (two-digit) code to be included. Returning to our example, we would not use information from men's shirts to predict the appearance of women's shirts. In Column 1 (three-digit) and 2 (two-digit), the Ecosystem density coefficient is statistically similar

to the original case reported in Table 2 in the main text, albeit with slightly lower AUCs.

Since our model describes the diversification of countries in terms of capability accumulation, we expect it to better predict the appearance of manufacturing products compared to natural resource or agricultural products. This is because the latter products tend to be present in countries mostly due to geographical or climate attributes. To test this, we split our sample into manufacturing products (including manufactures, chemicals, textiles, machinery and transport equipment) and non-manufacturing products (including agricultural raw materials, food, fuel, ores and metals) using a classification provided by the World Bank (http://wits.worldbank.org/data/public/CountryProfile-ProductMetadataforSITCRev2.xlsx). In Columns 3 (manufacturing) and 4 (non-manufacturing), we observe that the Ecosystem density is a significant predictor in both cases with a similar magnitude. However the AUC in manufacturing products is much higher compared to non-manufactured products.

In Columns 5 and 6, we split the products into high complexity (PCI above or equal to 0) and low complexity (PCI below 0) samples. We observe a significantly higher coefficient for the Ecosystem density variable for low complexity products, but the AUC is higher for high-complexity products.

In Columns 7 and 8, we split products into high ubiquity (ubiquity above or equal to median) and low ubiquity (ubiquity below median). Although the coefficients of the Ecosystem density variable are significantly different between two columns, the predictive power is similar as shown by the AUC.

Finally, in Columns 9 and 10, we split the sample into high export volume (export levels above or equal to median) and low volume (export levels below median). The coefficient for the Ecosystem density is lower for high export volume products, but the AUCs are similar in both cases. As in the case of countries described above, as the export volume of products increases, it becomes more difficult to satisfy the RCA-based criteria for a jump.

Supplementary Note 3.6 Different Specifications for Jump Definitions

In the construction of the Ecosystem matrix we allow countries to have multiple jumps to a single product in non-overlapping time periods. This could affect our results if there are countries with frequently repeated jumps to a wide variety of products. In Supplementary Table 3, we show the results for the Ecosystem density we obtain allowing a single jump only. The results are consistent with our main specification.

VARIABLES	(1)	(2)	(3)	(4)	(5)	(6)	(7)
Eco Density	9.3140***	11.3665***	8.5829***	9.8280***		10.3206***	8.8497***
	(0.3881)	(0.5455)	(0.4269)	(0.7729)		(0.4787)	(0.7420)
PS Density					1.7150***	-0.6333***	6.7179***
					(0.0986)	(0.1461)	(0.6473)
Constant	-10.3190***	-12.1730***	-9.3485***	-10.6180***	-1.9991***	-11.1619***	-9.9026***
	(0.3592)	(0.5169)	(0.4436)	(0.7461)	(0.0167)	(0.4321)	(0.7119)
Observations	49,278	49,121	39,346	39,206	49,278	49,278	39,206
Number of App.	1830	1830	1830	1830	1830	1830	1830
Country FE	No	Yes	No	Yes	No	No	Yes
Product FE	No	No	Yes	Yes	No	No	Yes
Pseudo R2	.061	.109	.109	.161	.015	.062	.169
AUC	.709	.770	.760	.811	.622	.712	.817

Robust standard errors in parentheses
*** $p<0.01$, ** $p<0.05$, * $p<0.1$

Supplementary Table 3: Allowing single jump per country-product instead of same country jumping on and off to same product multiple times.

We can also change the definition of jumps to be more stringent by requiring countries to be at least two consecutive years below the absence threshold and at least two consecutive years above the presence threshold. Supplementary Table 4 shows the results if we allow multiple jumps and Supplementary Table 5 shows the results if we allow single jumps with this more stringent definition. In both tables, the results do not change significantly compared to our main specification.

VARIABLES	(1)	(2)	(3)	(4)	(5)	(6)	(7)
Eco Density	6.4207***	6.3396***	7.5280***	6.7357***		6.1212***	5.2939***
	(0.5557)	(0.7732)	(0.6441)	(1.1945)		(0.6984)	(1.1468)
PS Density					1.9142***	0.2225	9.6053***
					(0.1690)	(0.2656)	(1.2384)
Constant	-8.0542***	-7.9571***	-9.1765***	-8.4042***	-2.5986***	-7.8176***	-8.8211***
	(0.5011)	(0.7241)	(0.7195)	(1.2077)	(0.0315)	(0.6056)	(1.1747)
Observations	37,031	30,082	14,184	11,490	37,031	37,031	11,490
Number of App.	351	351	351	351	351	351	351
Country FE	No	Yes	No	Yes	No	No	Yes
Product FE	No	No	Yes	Yes	No	No	Yes
Pseudo R2	.049	.086	.079	.128	.022	.049	.143
AUC	.709	.764	.740	.794	.665	.709	.810

Robust standard errors in parentheses
*** p<0.01, ** p<0.05, * p<0.1

Supplementary Table 4: Defining jumps to be from minimum two years absent to two years present allowing multiple jumps

VARIABLES	(1)	(2)	(3)	(4)	(5)	(6)	(7)
Eco Density	6.3881***	6.3956***	7.3766***	6.6964***		6.0720***	5.3279***
	(0.5539)	(0.7785)	(0.6325)	(1.2003)		(0.7032)	(1.1554)
PS Density					1.9229***	0.2339	9.6704***
					(0.1697)	(0.2682)	(1.2371)
Constant	-8.0136***	-7.9916***	-9.0055***	-8.3266***	-2.5959***	-7.7642***	-8.8336***
	(0.4988)	(0.7279)	(0.7133)	(1.2124)	(0.0316)	(0.6083)	(1.1877)
Observations	36,659	29,779	14,184	11,490	36,659	36,659	11,490
Number of App.	351	351	351	351	351	351	351
Country FE	No	Yes	No	Yes	No	No	Yes
Product FE	No	No	Yes	Yes	No	No	Yes
Pseudo R2	.048	.087	.078	.128	.022	.049	.143
AUC	.707	.764	.737	.793	.665	.707	.809

Robust standard errors in parentheses
*** p<0.01, ** p<0.05, * p<0.1

Supplementary Table 5: Defining jumps to be from minimum two years absent to two years present allowing only single jumps

Supplementary Note 3.7 Using different time periods to build the Ecosystem

Structural transformation is slow and countries need time to build their capabilities to jump into new products. On the other hand, the capability requirements of products might slowly change with the introduction of new technologies and production techniques. Therefore, we expect that the Ecosystem matrix will be optimally predictive for medium range time horizons. In addition,

the longer the data period used to build the Ecosystem matrix, the more jumps we observe and, hence, the better we expect the predictive power to be.

To test these hypotheses, we build different Ecosystem matrices by systematically varying the initial and final years of the trade data used. We require at least 10 years of data to build the Ecosystem matrices; hence, our initial years range from 1984 to 1999 and final years go from 1994 to 2009. In total we build 136 different Ecosystems and test their ability to predict jumps occurred between 2010 and 2016.

Supplementary Figure 11 shows AUC values for Ecosystem matrices constructed for different time horizons. As expected, almost all the periods ending close to our test period (i.e., 2010-2016) perform better. Even with periods ending in 2000, we have predictive power higher than 0.65. With the Product Space, this level of predictive power is never achieved no matter which year is used. As the final year of the time horizon to build the Ecosystem density or the Product Space density becomes further away from the test period, the predictive power declines. But even from the Ecosystem density built using transitions from 1984-1994, we achieve a comparable level of predictive power with respect to the best performing Product Space densities.

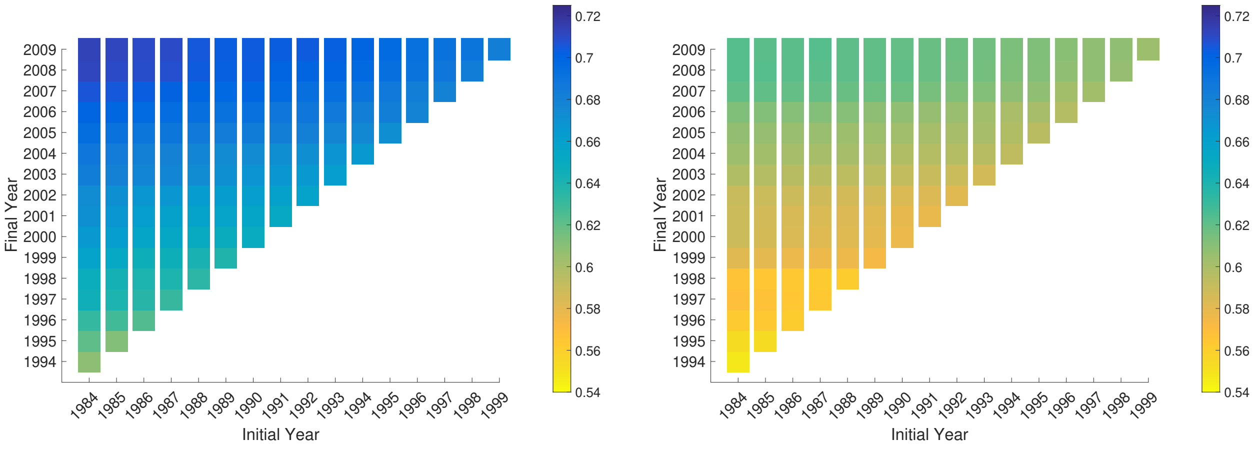

(a) Ecosystem (b) Product Space

Supplementary Figure 11: AUC results for various initial/final year combinations to build density variables. Each entry in the above matrices corresponds to the AUC value for the probit regressions with various year combinations to build the density variables to predict jumps occurred between 2010 and 2016. We required at least 10 years of data build the Ecosystem matix. The columns correspond to the start years, ranging from 1984 to 1999. The rows correspond to the final years, starting with 1994 and ending in 2009. In total, we have 136 distinct time periods. The corresponding Product Space density was built using the data from the final year of the period.

In Supplementary Figure 12, we repeat the same exercise, but instead of AUC metric, we use the pseudo-R^2 values to compare the predictive power of Ecosystem density and the Product Space density. As expected, the overall patterns are quite similar with Supplementary Figure 11.

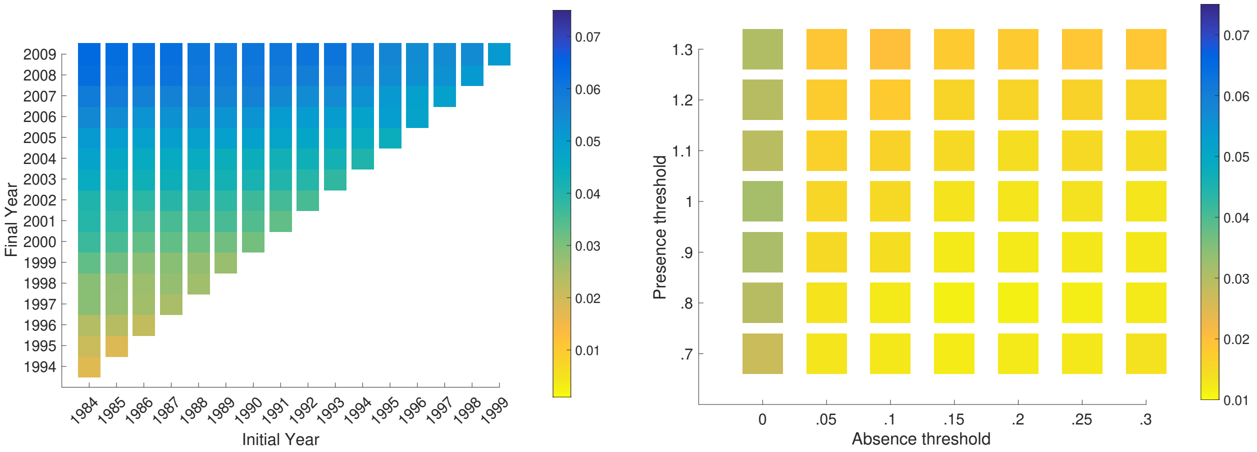

(a) Ecosystem (b) Product Space

Supplementary Figure 12: R^2 values for various initial/final year combinations to build density variables. Each entry in the above matrices corresponds to the pseudo-R^2 value for the probit regressions with various year combinations to build the density variables to predict jumps that occurred between 2010 and 2016. Please refer to the note under Supplementary Figure 11 for an explanation of each cell.

Supplementary Note 3.8 Using Revealed per-Capita Comparative Advantage (RpCA) instead of RCA

In our main specification, we use Balassa's Revealed Comparative Advantage[6] measure to determine whether a product is absent/present in a country. We can modify this measure to test our results' robustness. In particular, we can use a different measure to capture the intensity with which a country exports each product by computing its Revealed per-Capita Comparative Advantage (RpCA), which is a variant of RCA *adjusting for population*. The RpCA that a country has in a product is defined as the ratio between the share of total exports that the product represents in the country's export basket and its share of the global population. Congruently, we can also think of RpCA as the per capita export of the country in the product divided by the total per capita export in the world. A product is over-represented in a country's export basket if its RpCA is above a threshold.

Formally, if $X_{n,i}$ is equal to the export value of country n in product i and pop_n is the population

of country n, then the RpCA of country n in product i is defined as:

$$R_{n,i} = \frac{X_{n,i} / \sum_k X_{k,i}}{\text{pop}_n / \sum_k \text{pop}_k} \tag{1}$$

In order to estimate the presence (M) and jump (J) matrices, we can measure product presences and appearances via international export competitiveness captured by RpCA.

$$M_{n,i} = \begin{cases} 1 & \text{if } R_{n,i} > \tau_1 \text{ in } t \\ 0 & \text{otherwise} \end{cases} \tag{2}$$

An appearance of product i in country n is defined as:

$$J_{n,i} = \begin{cases} 1 & \text{if } R_{n,i} \leq \tau_0 \text{ in } t_0 \text{ and } R_{n,i} > \tau_1 \text{ in } t_1 \\ 0 & \text{otherwise} \end{cases} \tag{3}$$

Here, we use thresholds $\tau_0 = 0.05$ and $\tau_1 = 0.25$.

VARIABLES	(1)	(2)	(3)	(4)	(5)	(6)	(7)
Eco Density	19.5744***	13.2874***	22.8764***	8.3995***		13.5909***	15.0538***
	(0.6155)	(0.6920)	(0.7867)	(1.1687)		(0.6178)	(1.2246)
PS Density					1.7079***	1.0325***	24.7323***
					(0.0386)	(0.0465)	(1.4236)
Constant	-20.5204***	-14.9129***	-23.5303***	-10.1515***	-1.8384***	-14.9082***	-16.8238***
	(0.6014)	(0.6903)	(0.7923)	(1.1434)	(0.0123)	(0.5980)	(1.2012)
Observations	47,450	47,426	43,803	43,779	47,450	47,450	43,779
Number of App.	3153	3153	3153	3153	3153	3153	3153
Country FE	No	Yes	No	Yes	No	No	Yes
Product FE	No	No	Yes	Yes	No	No	Yes
Pseudo R2	.082	.160	.151	.229	.073	.101	.242
AUC	.731	.796	.790	.843	.723	.742	.850

Robust standard errors in parentheses
*** $p<0.01$, ** $p<0.05$, * $p<0.1$

Supplementary Table 6: Predicting future appearances based on RpCA. In this table all the densities are built using RpCA instead of RCA.

Supplementary Table 6 shows our results based on an Ecosystem matrix calculated using

RpCA. Appearances are also defined using RpCA. The results show that the Ecosystem based density is a better predictor of future product appearances relative to the Product Space.

Supplementary Note 4 Community Detection Algorithm

We use the community detection algorithm of Delvenne *et al.*[7] to investigate the two-digit reduced network and find that the network exhibits a modular structure composed of a number of well-defined communities. These communities are composed of groups of products that share similar capabilities, and are detected via an algorithm based on random walker dynamics. In essence, if we let a walker jump from node to node on a network with probability proportional to edge weight, eventually the walker will become trapped in 'regions' of the network exhibiting high internal connectivity, which correspond to node communities. The 'longer' the walker jumps, the larger the communities (s)he finds. Hence, a 'time' parameter enables us to control the scale at which communities are uncovered.

In practice, this algorithm works via optimisation of a stability function. In order to find the network partition that optimises this function, a greedy algorithm (the Louvain algorithm) is used. This algorithm is stochastic in the sense that it does not return an identical optimal partition on each run. We run this algorithm a large number of times and, to test the variation of these partitions, an information theoretic measure of overlap between each pair of partitions is calculated. The 'variation of information' measure is obtained by taking the average across all pairs. Hence, if the algorithm finds a large number of very similar optimal partitions, the variation of information will be low.

We ran this community detection algorithm on the directed network at two-digit level in Matlab with following parameters: $L = 10,000$ (i.e., number of optimisations of the Louvain algorithm to be done at each Markov time), $M = 1,000$ (the top M partitions among the L given at each Markov time by the L Louvain optimisations will be used to compute the variation of information), enforcing the calculation of the full stability and teleportation probability of 0.

Supplementary Figure 13 shows the output of the stability algorithm. The left panel shows the number of communities and as the right panel shows the variation of information. We observe a

low variation of information for all Markov times below time 14, indicating stable partitions for this time parameter range. We selected the community corresponding to Markov Time 8 which has 5 distinctive communities and two singleton products. We merge these singleton products into the five communities based on their similarity to community members.

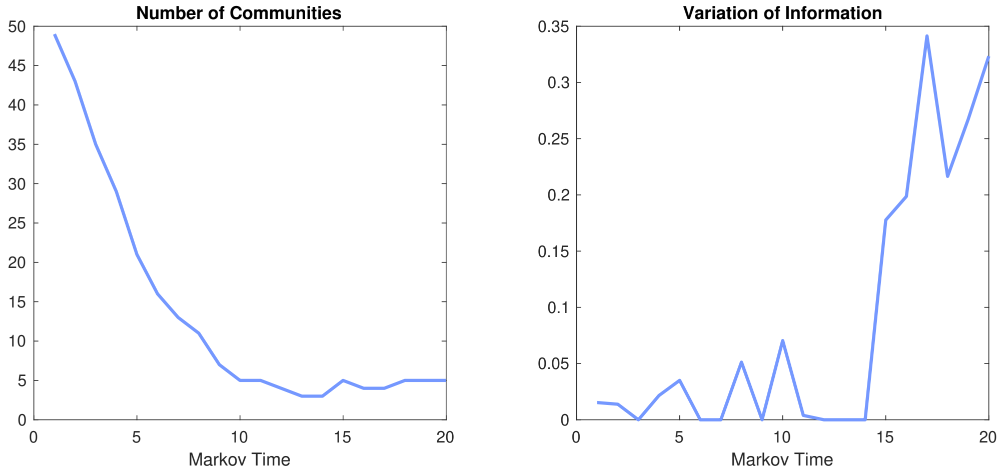

Supplementary Figure 13: Output of Stability Algorithm. The left panel shows the number of communities for different time variables while the right panel shows the corresponding variance of information. The x-axis correspond to different time parameters. We sampled the Markov time from $10^{-0.15}$ to 10^2 in 20 equal log-spaced steps using Matlab's logspace command.

Supplementary Note 5 Country Presences in Communities

In the main text, we show countries colored by the community in which they have the greatest share of products present. But not all communities have the same size: Community 1 has 98 products, 2 has 111 products, 3 has 179 products, 4 has 91 products and 5 has 272 products in it. How can we normalise for the community size, and how can we tell whether a country has significant presence in a community?

Here, we use the logic behind RCA for matching countries to the communities, which can also be thought of as the likelihood ratio. Formally, we can calculate the likelihood ratio as the share of products present in a country belonging to a community divided by the share of total products in that community. Let P_{nc} show the number of products present in country n that belong to community c. Let, P_c denote the number of products in community c. Hence, we can write the

Likelihood ratio (LR) as:

$$\mathrm{LR}_{nc} = \frac{P_{nc} / \sum_c P_{nc}}{P_c / \sum_c P_c}$$

An $\mathrm{LR}_{nc} > 1$ implies that the country n has more products in community c than expected.

Supplementary Figure 14a shows countries that have a higher number of products than their expected share in Community 1, which consists of sectors like furniture, building materials, manufactures of metals and nonmetal minerals, misc edible and live animals. In addition to European and North American countries, China, Japan, India, Malaysia, South Africa, Egypt, Turkey, UA, Brazil and Colombia have a significant presence in this community.

Supplementary Figure 14b shows countries that have a higher number of products than their expected share in Community 2, which consists of sectors mostly associated with natural resources. The distribution of countries also follow the natural resource wealth of countries.

Supplementary Figure 14c shows countries that have a higher number of products than their expected share in Community 3, which consists of sectors like machinery, medicine and pharmaceuticals, electronics and office machinery. These are sophisticated products and countries that have an over-abundance in this community are in North America, Europe and East Asia.

Supplementary Figure 14d shows countries that have a higher number of products than their expected share in Community 4, which consists of sectors like transport equipment, paper related, general industrial machinery and meat preparation. The pattern of countries that are over abundant in this community is very similar to community 1 with Egypt, Tunisia and UAE dropping out, but Indonesia and New Zealand joining.

Supplementary Figure 14e shows countries that have a higher number of products than their expected share in Community 5, which consists of mostly primary sectors. These are mostly developing countries with notable additions like Eastern European countries, Spain and Australia. Interestingly, most East Asian countries like China are not over-represented in this community.

Finally, Supplementary Figure 14f shows countries coloured by the community in which they have the highest likelihood ratio (LR). Notably, countries that have their highest LR in Community 3 (coloured blue) are East Asian countries like China, Japan, Singapore and Malaysia. European and North American countries follow a different pattern, and are often associated with Commu-

nity 4 (green).

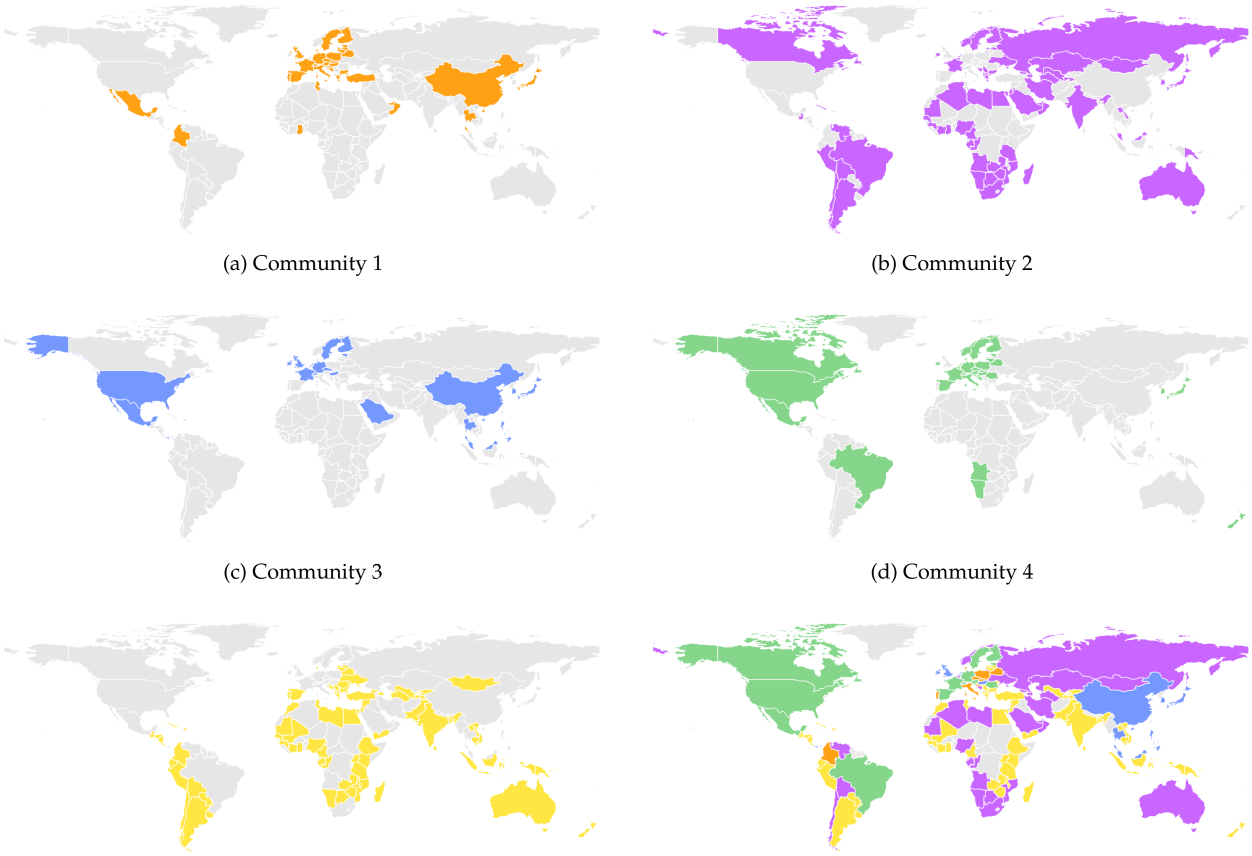

(a) Community 1

(b) Community 2

(c) Community 3

(d) Community 4

(e) Community 5

(f) Community by Max

Supplementary Figure 14: Over-represented communities by country. Over-representation is determined by the likelihood ratio of observing products within a community in a country.

Supplementary Note 6 Additional Figures

Supplementary Figure 15 replicates a number of sub-plots in Figures 1 and 3 of the main text. Here our Ecosystem size and input vectors are computed using the weighted Ecosystem entries rather than binary counting of non-zero entries. We observe relationships between the Ecosystem size and input with product ubiquity and Product Complexity Index[8] consistent with those shown in the main text.

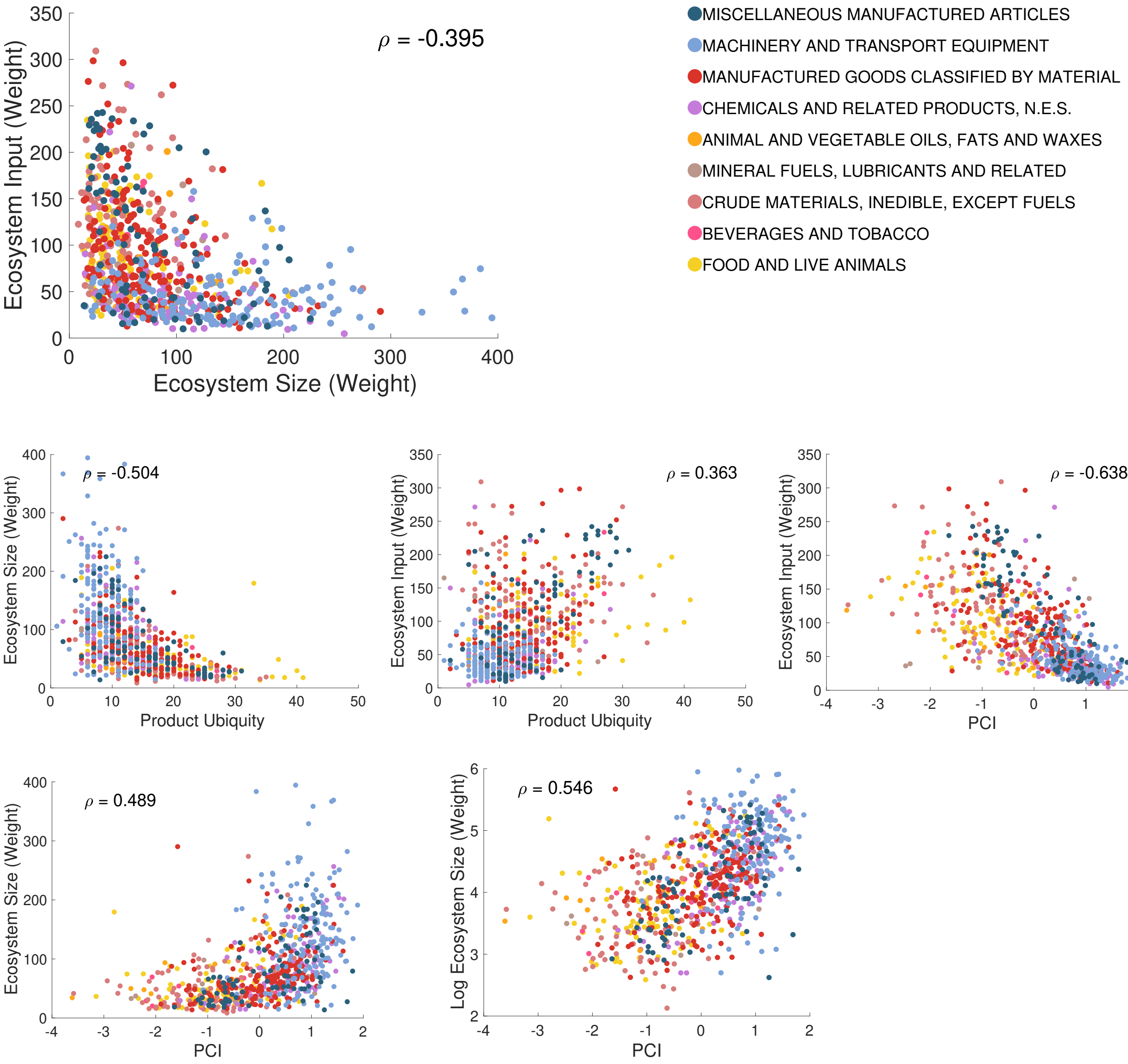

Supplementary Figure 15: Ecosytem Network properties for the Weighted Network. Here, we use the weighted Ecosystem matrix instead of the thresholded version.

References

[1] Duda, R. O., Hart, P. E. & Stork, D. G. *Pattern classification* (John Wiley & Sons, 2000).

[2] Hidalgo, C. A., Klinger, B., Barabási, A.-L. & Hausmann, R. The product space conditions the development of nations. *Science* **317**, 482–487 (2007).

[3] Fawcett, T. An introduction to roc analysis. *Pattern Recognition Letters* **27**, 861–874 (2006).

[4] Hosmer Jr, D. W., Lemeshow, S. & Sturdivant, R. X. *Applied logistic regression*, vol. 398 (John Wiley & Sons, 2013).

[5] Hanley, J. A. & McNeil, B. J. The meaning and use of the area under a receiver operating characteristic (roc) curve. *Radiology* **143**, 29–36 (1982).

[6] Balassa, B. Trade liberalisation and "revealed" comparative advantage 1. *The Manchester School* **33**, 99–123 (1965).

[7] Delvenne, J.-C., Yaliraki, S. N. & Barahona, M. Stability of graph communities across time scales. *Proceedings of the National Academy of Sciences* **107**, 12755–12760 (2010).

[8] Hidalgo, C. A. & Hausmann, R. The building blocks of economic complexity. *Proceedings of the National Academy of Sciences of the United States of America* **106**, 10570–10575 (2009).
